# A streamlined synthetic approach to the truncated linear trisaccharide fragment of QS-21

**DOI:** 10.3389/fchem.2025.1650302

**Published:** 2025-09-23

**Authors:** Jhe-Sian Lin, Zheng-Hao Tzeng, Jasper S. Dumalaog, Shang-Cheng Hung

**Affiliations:** 1 Institute of Biochemistry and Molecular Biology, National Yang Ming Chiao Tung University, Taipei, Taiwan; 2 Genomics Research Center, Academia Sinica, Taipei, Taiwan; 3 Department of Chemistry, National Tsing Hua University, Hsinchu, Taiwan; 4 Department of Applied Science, National Taitung University, Taitung, Taiwan; 5 Department of Chemistry, National Cheng Kung University, Tainan, Taiwan

**Keywords:** QS-21, linear trisaccharide, glycosylation, carbohydrate chemistry, vaccine adjuvant

## Abstract

QS-21, a potent immunostimulatory saponin obtained from *Quillaja saponaria* Molina, a soapbark tree native to Chile, has undergone extensive study for its broad application as a vaccine adjuvant against various infectious diseases and cancers. The structure of QS-21, which features a linear oligosaccharide moiety, provides a critical attachment site for both the labile acyl side chain and the distinctive sugar unit that defines each major saponin variant. In this study, we present an efficient synthetic approach to the truncated linear trisaccharide fragment of QS-21, circumventing the challenges associated with the synthesis of the rare sugar D-fucose. The synthesis of this linear trisaccharide enables streamlined access to a homogeneous QS-21.

## 1 Introduction

Adjuvants play a crucial role in enhancing vaccine effectiveness by stimulating the immune system to produce a robust response (Reed et al., 2013). Among vaccine adjuvants, QS-21 stands out for its potent immunostimulatory properties. It is a natural saponin derived from the bark of the Chilean soapbark tree, *Quillaja saponaria* (QS) Molina, which contains over 100 structurally related QS saponins due to its diverse composition (Reed et al., 2023). QS-21 is identified as the 21st fraction of 22 obtained from the reverse-phase high-performance liquid chromatography (HPLC) of the semi-purified QS extract with potent adjuvant activity, hence its name (Kensil et al., 1991; Martin et al., 2024). Currently, QS-21 has been the most extensively studied saponin adjuvant for over 28 years (Ragupathi et al., 2011). It has been shown to stimulate both cellular (Th1) and humoral (Th2) immune responses, making it highly effective in enhancing immunogenicity (Fernández-Tejada et al., 2014; Pink and Kieny, 2004). In 2017, QS-21 was first licensed for human use as a vaccine adjuvant, specifically for the herpes zoster vaccine Shingrix^®^ (Lacaille-Dubois, 2019). Extensive clinical studies have demonstrated its strong immunostimulatory effects, significantly improving vaccine efficacy against a range of infectious diseases and cancers (Garçon and Van Mechelen, 2011; Gin and Slovin, 2011).

**FIGURE 1:**
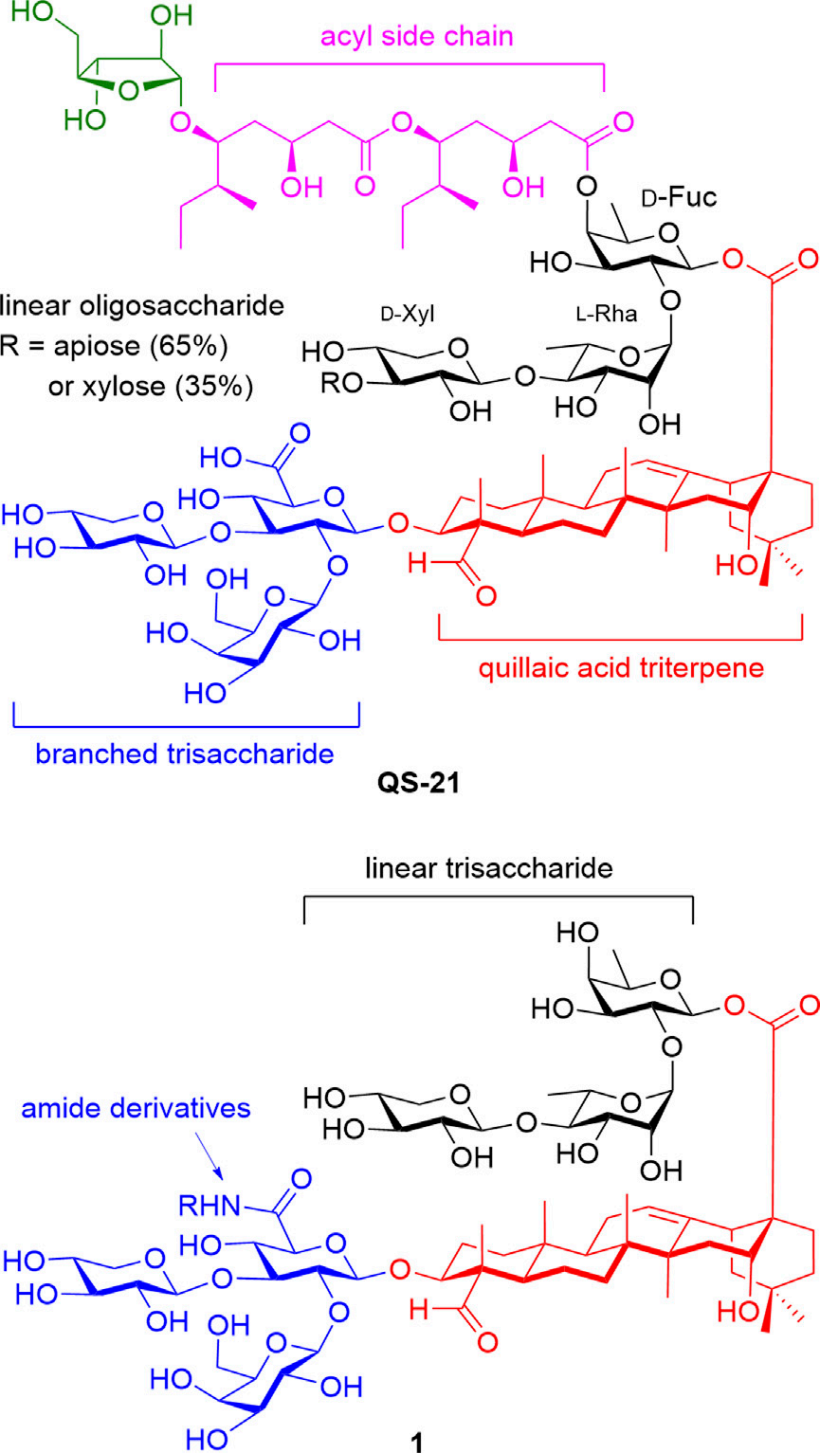
Structure of QS-21 and its saponin conjugate **1**.

The structure of QS-21, as illustrated in Figure 1, consists of a quillaic acid triterpene attached to branched trisaccharide and linear oligosaccharide moieties. QS-21 is a mixture of two isomers, apiose- (65%) and xylose-containing (35%) oligosaccharides, attached to the D-xylose ring of the linear trisaccharide group. These two isomers were found to have similar adjuvanticity and toxicity (Ragupathi et al., 2010). A labile acyl side chain containing an arabinofuranose ring is connected to the D-fucose ring of the linear oligosaccharide. Despite its immunostimulatory properties, the instability of the acyl side chain and the challenges associated with its low-yielding purification limit the broader application of QS-21. Moreover, the loss of the lipophilic side chain due to the hydrolysis of the ester linkage results in the loss of its adjuvanticity (Marciani et al., 2001).

Numerous efforts have been undertaken to synthesize analogs of QS-21 with comparable or enhanced potency to mitigate these challenges. Wang et al. (2005) achieved the pioneering total synthesis of QS-21 and its definitive structural characterization. Additionally, they conducted an efficient semi-synthesis of various QS-21 variants to develop immunoadjuvants with improved chemical stability (Chea et al., 2012; Fernández-Tejada et al., 2016). Liang et al. (2020) patented the synthesis of saponin conjugates, including the truncated QS-21 moiety **1** (Figure 1), which enhances efficacy in both humoral and cell-mediated immunity. The structure of **1** comprises a truncated linear trisaccharide featuring a *β*-D-Xyl*p-(*1→4)*-α*-L-Rha*p*-(1→2)-*β*-D-Fuc*p* moiety linked to the quillaic acid triterpene.

In this study, we present an efficient and streamlined synthesis of the linear trisaccharide moiety derived from saponin conjugate **1**, offering an alternative approach to the synthesis of these QS-21 oligosaccharide units. Two complementary strategies, pre- and post-glycosylation deoxygenation, were explored to address key synthetic challenges. These included the efficient incorporation of the rare sugar D-fucose and the stereoselective construction of oligosaccharides featuring 1,2-*trans*-glycosidic bonds, both of which are pivotal for constructing the biologically relevant glycan structure.

## 2 Materials and methods

### 2.1 General procedures

All moisture-sensitive reactions were carried out under an N_2_ atmosphere in flame-dried glassware. Solvents such as dichloromethane (CH_2_Cl_2_), acetonitrile (CH_3_CN), and tetrahydrofuran (THF) were distilled using a purification system with activated Al_2_O_3_. All commercially obtained reagents were used without additional purification, unless specified otherwise. All distilled water used was purified using a Milli-Q system. Prior to all glycosylations, the starting materials were thoroughly dried under high vacuum in a desiccator. Thin-layer chromatography (TLC) analysis was conducted on Silica Gel 60G F_254_ glass plates (0.25 mm, E. Merck from Germany). TLC analysis was performed with visualization under ultraviolet light (UV-254 nm), and staining was carried out by spraying with a solution of Hanessian’s reagent containing Ce(NH_4_)_2_(NO_3_)_6_, (NH_4_)_6_Mo_7_O_24_, and H_2_SO_4_ in water, followed by heating on a hot plate. Flash column chromatography was conducted on Silica Gel 60 (230–400 mesh, E. Merck).

Specific rotations were measured at ambient temperature using a HORIBA SEPA-300 High-Sensitive Polarimeter from Kyoto, Japan at 589 nm (sodium D line) and reported in 10^−1^⋅deg⋅cm^2^⋅g^–1^, with sample concentrations given in g⋅dL^–1^. IR spectra were recorded on KBr plates using a PerkinElmer Spectrum 100 FT-IR Spectrometer from Waltham, Massachusetts, USA. 1D and 2D NMR spectra were acquired using a Bruker Avance III 600 MHz spectrometer from Billerica, Massachusetts, USA at ambient temperature. Data were recorded as follows: chemical shift in ppm from the solvent resonance used as the internal standard (CDCl_3_ at 7.26 ppm), multiplicity (s, singlet; d, doublet; t, triplet; q, quartet; m, multiplet), coupling constant in Hz, and integration. ^13^C NMR spectra were obtained using a 150 MHz spectrometer, and chemical shifts were recorded in ppm relative to the solvent resonance used as the internal standard (CDCl_3_ at 77.0 ppm). Mass spectra were acquired using an ESI Finnigan LCQ Mass Spectrometer (Thermo Finnigan from Waltham, Massachusetts, USA), performed at the Genomics Research Center.

### 2.2 Synthetic procedures and characterization data

#### 2.2.1 Benzyl 2,3,4,6-tetra-*O*-acetyl-*β*-D-galactopyranoside (**9**)

To a stirred suspension of **14** (2.0 g, 5.12 mmol) in BnOH (1.1 mL), BF_3_•Et_2_O (2.6 mL, 20.5 mmol) was added at 0 °C under an N_2_ atmosphere. Upon completion of the reaction after 16 h, the mixture was diluted with CH_2_Cl_2_, washed with H_2_O and brine, dried over MgSO_4_, and then concentrated *in vacuo*. The residue was purified by column chromatography (silica gel; ethyl acetate/hexane = 1/4) to afford **9** (2.24 g, 75%). The IR spectrum (thin film) showed absorption bands at *ν* 2,924, 1,749, 1,369, and 1,221 cm^–1^. The ^1^H NMR spectrum (600 MHz, CDCl_3_) exhibited signals at δ 7.35–7.22 (m, 5H, Ar-H), 5.36 (dd, *J* = 3.5, 1.2 Hz, 1H, H-4), 5.26 (dd, *J* = 10.5, 7.9 Hz, 1H, H-2), 4.96 (dd, *J* = 10.5, 3.5 Hz, 1H, H-3), 4.89 (d, *J* = 12.4 Hz, 1H, Ar-CH_2_), 4.61 (d, *J* = 12.3 Hz, 1H, Ar-CH_2_), 4.49 (d, *J* = 8.0 Hz, 1H, H-1), 4.22–4.09 (m, 2H, H-6), 3.86 (td, *J* = 6.7, 1.2 Hz, 1H, H-5), 2.14 (s, 3H, CH_3_), 2.04 (s, 3H, CH_3_), 1.99 (s, 3H, CH_3_), and 1.95 (s, 3H CH_3_). The ^13^C NMR spectrum (150 MHz, CDCl_3_) displayed resonances at δ 170.4 (C), 170.3 (C), 170.1 (C), 169.4 (C), 136.7 (C), 128.4 (CH), 127.9 (CH),127.7 (CH), 99.8 (CH), 70.9 (CH), 70.8 (CH_2_), 70.7 (CH), 68.8 (CH), 67.0 (CH), 61.2 (CH_2_), 20.8 (CH_3_), 20.7 (CH_3_), 20.7 (CH_3_), and 20.6 (CH_3_). High-resolution mass spectrometry (HRMS) (ESI) analysis showed a peak at *m/z* 456.1869, which is consistent with the calculated value of *m/z* 456.1864 for C_21_H_26_O_10_NH_4_ ([M + NH_4_]^+^).

#### 2.2.2 Benzyl 3,4-*O*-isopropylidene-*β*-D-galactopyranoside (**8**)

Compound **9** (493.3 mg, 1.83 mmol) and MeOH (6 mL) were added to the flask, and the mixture was stirred at 0 °C for 30 min. NaOMe (19.4 mg, 0.36 mmol) was slowly added to the reaction mixture in 10 mg portions at 10 min intervals, and the mixture was then allowed to warm to room temperature (RT) and react overnight. The reaction progress was monitored by TLC (ethyl acetate/hexane = 1/2). The reaction mixture was neutralized with Dowex® 50W × 8 resin to pH 5–6, filtered directly, and concentrated (45 °C, below 25 mbar) to obtain an off-white solid. The solid was vacuum-dried for over 16 h and used directly in the following steps without additional purification.

To a stirred suspension of the crude intermediate in anhydrous CH_3_CN (6 mL), 2,2-dimethoxypropane (2,2-DMP, 1.35 mL, 3.65 mmol) and 10-camphorsulfonic acid (CSA, 0.13 g, 0.55 mmol) were added at RT under an N_2_ atmosphere. Upon completion of the reaction after 30 min, the mixture was diluted with CH_2_Cl_2_, washed with H_2_O and brine, dried over MgSO_4_, and then concentrated under reduced pressure. The residue was purified by column chromatography (silica gel; ethyl acetate/hexane = 3/2) to yield **8** (301 mg, 53%). The specific rotation was [α]^29^_D_ +6.16 (*c* 0.6, CHCl_3_). The IR spectrum (thin film) showed absorption bands at *ν* 3,400, 2,919, 1,454, and 1,040 cm^–1^. The ^1^H NMR spectrum (600 MHz, CDCl_3_) exhibited signals at δ 7.34 (d, *J* = 4.4 Hz, 4H, Ar-H), 7.30 (ddt, *J* = 8.4, 6.9, 3.6 Hz, 1H, Ar-H), 4.90 (d, *J* = 11.7 Hz, 1H, Ar-CH_2)_, 4.64 (d, *J* = 11.7 Hz, 1H, Ar-CH_2_), 4.27 (d, *J* = 8.2 Hz, 1H, H-1), 4.13 (dd, *J* = 5.5, 2.1 Hz, 1H, H-5), 4.07 (dd, *J* = 7.4, 5.5 Hz, 1H, H-3), 3.97 (dd, *J* = 12.8, 8.2 Hz, 1H, H-6a), 3.82 (qd, *J* = 5.4, 4.2, 2.2 Hz, 2H, 4-H, H-6b), 3.60 (t, *J* = 7.8 Hz, 1H), 2.43 (s, 1H, 2-OH), 2.07–2.02 (m, 1H, 6-OH), 1.59 (s, 3H, CH_3_), and 1.32 (s, 3H, CH_3_). The ^13^C NMR spectrum (150 MHz, CDCl_3_) displayed resonances at δ 136.6 (C), 128.4 (CH), 128.1 (CH), 127.9 (CH), 110.3 (C), 101.1 (CH), 78.6 (CH), 73.7 (CH), 73.5 (CH), 73.3 (CH), 71.2 (CH_2_), 62.3 (CH_2_), 27.9 (CH_3_), and 26.1 (CH_3_). HRMS (ESI) analysis showed a peak at *m/z* 328.1757, which is consistent with the calculated value of *m/z* 328.1755 for C_16_H_22_O_6_NH_4_ ([M + NH_4_]^+^).

#### 2.2.3 Benzyl 6-*O*-acetyl-3,4-*O*-isopropylidene-*β*-D-galactopyranoside (**5**)

To a stirred suspension of **8** (76 mg, 0.25 mmol) in anhydrous CH_2_Cl_2_ (0.7 mL), Et_3_N (0.23 g, 1.0 mmol) and Ac_2_O (24 μL, 0.26 mmol) were added at 0 °C under an N_2_ atmosphere. Upon completion of the reaction after 2 h, the mixture was diluted with CH_2_Cl_2_, washed with H_2_O and brine, dried over MgSO_4_, and then concentrated under reduced pressure. The residue was purified by column chromatography (silica gel; ethyl acetate/hexane = 1/2) to yield **5** (66.9 mg, 76%). The specific rotation was [α]^28^_D_ +12.1 (*c* 1.0, CHCl_3_). The IR spectrum (thin film) showed absorption bands at *ν* 3,454, 2,987, 1,741, 1,242, and 1,076 cm^–1^. The ^1^H NMR spectrum (600 MHz, CDCl_3_) exhibited signals at δ 7.34 (d, *J* = 4.3 Hz, 4H, Ar-H), 7.30 (q, *J* = 4.4 Hz, 1H, Ar-H), 4.91 (d, *J* = 11.5 Hz, 1H, Ar-CH_2_), 4.60 (d, *J* = 11.6 Hz, 1H, Ar-CH_2_), 4.41–4.35 (m, 2H, H-6), 4.21 (d, *J* = 8.4 Hz, 1H, H-1), 4.13–4.09 (m, 1H, H-4), 4.04 (t, *J* = 6.5 Hz, 1H, H-3), 3.94 (t, *J* = 6.0 Hz, 1H, H-5), 3.60 (t, *J* = 7.9 Hz, 1H, H-2), 2.45 (s, 1H, 2-OH), 2.10 (d, *J* = 1.5 Hz, 3H, CH_3_), 1.50 (s, 3H, CH_3_), and 1.32 (s, 3H, CH_3_). The ^13^C NMR spectrum (150 MHz, CDCl_3_) displayed resonances at δ 170.8 (C), 136.7 (C), 128.5 (CH), 128.3 (CH), 128.1 (CH), 110.6 (C), 101.8 (CH), 78.6 (CH), 73.5 (CH), 73.4 (CH), 71.2 (CH), 70.9 (CH_2_), 63.5 (CH_2_), 28.1 (CH_3_), 26.3 (CH_3_), and 20.9 (CH_3_). HRMS (ESI) analysis showed a peak at *m/z* 370.1861, which is consistent with the calculated value of *m/z* 370.1860 for C_18_H_24_O_7_NH_4_ ([M + NH_4_]^+^).

#### 2.2.4 Benzyl 6-*O*-methanesulfonyl-3,4-*O*-isopropylidene*-β*-D-galactopyranoside (**20**)

To a stirred suspension of **8** (60 mg, 0.194 mmol) in anhydrous CH_2_Cl_2_ (2.4 mL), Et_3_N (27.1 μL, 0.194 mmol) and MsCl (15.1 μL, 0.388 mmol) were added at 0 °C under an N_2_ atmosphere. Upon completion of the reaction after 1 h, the mixture was diluted with CH_2_Cl_2_, washed with H_2_O and brine, dried over MgSO_4_, and then concentrated under reduced pressure. The residue was purified by column chromatography (silica gel; ethyl acetate/hexane = 1/3) to afford **20** (47.5 mg, 63%). The specific rotation was [α]^28^_D_ +7.80 (*c* 0.5, CHCl_3_). The IR spectrum (thin film) showed absorption bands at *ν* 3,492, 2,922, 1,712, 1,355, and 1,073 cm^–1^. The ^1^H NMR spectrum (600 MHz, CDCl_3_) exhibited signals at δ 7.34 (s, 3H, Ar-H), 7.38–7.27 (m, 2H, Ar-H), 4.90 (d, *J* = 11.6 Hz, 1H, Ar-CH_2_), 4.62 (d, *J* = 11.6 Hz, 1H, Ar-CH_2_), 4.51–4.42 (m, 2H, H-6), 4.25 (d, *J* = 8.3 Hz, 1H, H-1), 4.13 (dd, *J* = 5.6, 2.3 Hz, 1H, H-4), 4.11–4.04 (m, 2H, 3-H, H-5), 3.60 (td, *J* = 7.9, 1.9 Hz, 1H, H-2), 3.04 (s, 3H, SO_2_CH_3_), 2.40 (d, *J* = 2.4 Hz, 1H, OH), 1.50 (s, 3H, CH_3_), and 1.32 (s, 3H, CH_3_). The ^13^C NMR spectrum (150 MHz, CDCl_3_) displayed resonances at δ 136.5 (C), 128.61 (CH), 128.28 (CH), 128.24 (CH), 110.7 (C), 100.83 (CH), 78.6 (CH), 73.4 (CH), 73.0 (CH), 71.2 (CH_2_), 68.7 (CH_2_), 37.38 (CH_3_), 28.00 (CH_3_), and 26.31 (CH_3_). HRMS (ESI) analysis showed a peak at *m/z* 406.1535, which is consistent with the calculated value of *m/z* 406.1530 for C_17_H_24_O_8_SNH_4_ ([M + NH_4_]^+^).

#### 2.2.5 Benzyl 6-deoxy-6-iodo-3,4-*O*-isopropylidene-*β*-D-galactopyranoside (**21**)

To a solution of **20** (20.2 mg, 0.059 mmol) in DMF (2.0 mL), tetrabutylammonium iodide (TBAI) (19.2 mg, 0.059 mmol) and KI (29.7 mg, 0.179 mmol) were added. The solution was stirred at 120 °C for 26 h, then cooled to RT, diluted with water, and extracted using ethyl acetate. The combined organic layer was washed with saturated aqueous Na_2_S_2_O_3_ and brine and then dried over anhydrous Na_2_SO_4_. The organic layer was evaporated, and the residue was purified by silica gel chromatography (silica gel; ethyl acetate/hexane = 1/3) to afford **21** (24.8 mg, 72%). The specific rotation was [α]^28^_D_ +12.1 (*c* 0.5, CHCl_3_). The IR spectrum (thin film) showed absorption bands at *ν* 3,432, 2,920, and 1,065 cm^–1^. The ^1^H NMR spectrum (600 MHz, CDCl_3_) exhibited signals at δ 7.40–7.32 (m, 4H, Ar-H), 7.34–7.27 (m, 1H, Ar-H), 4.95 (d, *J* = 11.6 Hz, 1H, Ar-CH_2_), 4.65 (d, *J* = 11.7 Hz, 1H, Ar-CH_2_), 4.27 (dd, *J* = 5.5, 2.3 Hz, 1H, H-4), 4.22 (d, *J* = 8.3 Hz, 1H, H-1), 4.05 (dd, *J* = 7.4, 5.4 Hz, 1H, H-3), 3.88 (ddd, *J* = 7.3, 6.7, 2.3 Hz, 1H, H-5), 3.59 (ddd, *J* = 8.3, 7.4, 2.3 Hz, 1H, H-2), 3.43 (dd, *J* = 7.0, 0.9 Hz, 2H, H-6a, H-6b), 2.34 (d, *J* = 2.3 Hz, 1H, OH), 1.50 (s, 3H, CH_3_), and 1.34 (d, *J* = 0.8 Hz, 3H, CH_3_). The ^13^C NMR spectrum (150 MHz, CDCl_3_) displayed resonances at δ 136.6 (C), 128.6 (CH), 128.5 (CH), 128.2 (CH), 110.29 (C), 100.62 (CH), 78.7 (CH), 74.0 (CH), 73.8 (CH), 73.5 (CH), 70.79 (CH_2_), 28.1 (CH_3_), 26.2 (CH_3_), and 1.86 (CH_2_). HRMS (ESI) analysis showed a peak at *m/z* 438.0777, which is consistent with the calculated value of *m/z* 438.0772 for C_16_H_21_IO_5_NH_4_ ([M + NH_4_]^+^).

#### 2.2.6 Benzyl 3,4-*O*-isopropylidene-*β*-D-fucopyranoside (**10**)

To a solution of **21** (10.1 mg, 0.024 mmol) in THF/MeOH = 10/1 (2 mL), Pd(OH)_2_/C (20% wt, 10.1 mg) was added. The resulting suspension was stirred under H_2(g)_ at room temperature and atmospheric pressure for 6 h. The reaction mixture was filtered through Celite and concentrated under reduced pressure. The residue was purified by silica gel chromatography (silica gel; ethyl acetate/hexane = 1/3) to afford **10** (6.72 mg, 95%). The specific rotation was [α]^29^_D_ −28.5 (*c* 0.1, CHCl_3_). The IR spectrum (thin film) showed absorption bands at *ν* 3,445, 2,926, and 1,071 cm^–1^. The ^1^H NMR spectrum (600 MHz, CDCl_3_) displayed signals at δ 7.37–7.31 (m, 4H, Ar-H), 7.29 (ddt, *J* = 8.8, 6.6, 3.0 Hz, 1H, Ar-H), 4.92 (dd, *J* = 11.6, 1.6 Hz, 1H, Ar-CH_2_), 4.57 (dd, *J* = 11.6, 1.8 Hz, 1H Ar-CH_2_), 4.21 (dd, *J* = 8.3, 1.6 Hz, 1H, H-1), 4.03–3.98 (m, 1H, H-3), 4.00–3.96 (m, 1H, H-4), 3.84 (qd, *J* = 6.6, 3.5 Hz, 1H, H-5), 3.61–3.55 (m, 1H, H-2), 2.35 (s, 1H, OH), 1.52 (s, 3H, CH_3_), 1.43 (dd, *J* = 6.6, 1.7 Hz, 3H, H-6), and 1.34 (s, 3H, CH_3_). The ^13^C NMR spectrum (150 MHz, CDCl_3_) exhibited resonances at δ 137.0 (C), 128.5 (CH), 128.3 (CH), 128.0 (CH), 109.9 (C), 100.9 (CH), 78.7(CH), 76.3 (CH), 73.6 (CH), 70.8 (CH_2_), 69.2 (CH), 28.2 (CH_3_), 26.3 (CH_3_), and 16.6 (CH_3_). HRMS (ESI) analysis showed a peak at *m/z* 312.1810, which is consistent with the calculated value of *m/z* 312.1805 for C_16_H_22_O_5_NH_4_ ([M + NH_4_]^+^).

#### 2.2.7 2,3,4-Tri-*O*-acetyl-*α,β*-D-xylopyranoside (**12**)

A 12-L reaction flask and a 1-L addition funnel were dried and allowed to cool to room temperature. D-Xylose (500 g, 3.33 mol), Et_3_N (2.8 L, 19.9 mol), and DMAP (40.7 g, 0.33 mol) were added to the flask, and the mixture was cooled to 0 °C and stirred for 30 min. Ac_2_O (1.57 L, 16.6 mol) was placed in an addition funnel and added dropwise over approximately 1 h. The mixture changed from pale yellow and turbid to dark brown while remaining turbid. The reaction was stirred for approximately 3 h. The reaction progress was monitored by TLC (ethyl acetate/hexane = 1/1). The reaction mixture was quenched by pouring into 2 L of ice water, extracted with ethyl acetate, and washed with saturated aqueous NaHCO_3_ solution. The organic layer was dried over MgSO_4_, filtered, concentrated (45 °C, below 20 mbar), and used directly in the subsequent steps. Mechanical stirring was set up for a 20-L reaction flask. Compound **12** and THF were added to the flask, and the mixture was cooled to 0 °C and stirred for 30 min. BnNH_2_ (610.2 g, 5.59 mol) was added dropwise using a 1-L addition funnel. The mixture was allowed to warm to room temperature and react overnight. The reaction progress was monitored by TLC (ethyl acetate/hexane = 1/1). The reaction mixture was quenched with 1 N HCl_(aq)_ and neutralized to a pH of 0–2. The mixture was extracted twice with ethyl acetate/brine, dried over MgSO_4_, filtered, and concentrated (45 °C, below 30 mbar). The crude product was then subjected to 151G3 flash column chromatography in three batches. The 151G3 flash column (diameter: 18 cm; height: 20 cm; volume: approximately 5 L) was loaded with crude product (approximately 300 g) that had been premixed with silica gel (approximately 300 g) in an ethyl acetate/hexane = 1/2 mixture. The column was packed with 2 L of silica gel, followed by the addition of the crude solution. The column was eluted with an ethyl acetate/hexane = 1/2 mixture (approximately 3 L per 8 fractions). The eluent was concentrated (45 °C, below 15 mbar) and allowed to stand overnight, yielding a precipitate. The solid was washed with hexane, dried under vacuum, and afforded a white solid (561 g) with a yield of 62% (over two steps). The specific rotation was [α]^28^_D_ +58.4 (*c* 1.0, CHCl_3_). The IR spectrum (thin film) showed absorption bands at *ν* 3,393, 2,947, 1,757, 1,230, and 1,052 cm^–1^.

#### 2.2.8 *p*-Methylphenyl 2,3-*O*-isopropylidene-1-thio-*α*-L-rhamnopyranoside (**7**)

A 5-L reaction flask was dried and allowed to cool to room temperature. Compound **13** (300 g, 0.757 mol) and MeOH (3 L) were added to the flask, and the mixture was stirred at 0 °C for 30 min. NaOMe (40.9 g, 0.757 mol) was slowly added to the reaction mixture in 10 g portions at 10 min intervals, and the mixture was allowed to warm to room temperature and react overnight. The reaction progress was monitored by TLC (ethyl acetate/hexane = 2/1). The reaction mixture was neutralized with Dowex® 50W × 8 resin (prewashed twice with MeOH, approximately 330 g) to a pH of 5–6, filtered directly, and concentrated (45 °C, below 25 mbar) to obtain an off-white solid. The solid was dried under vacuum for over 16 h and then used directly in the subsequent steps without further purification.

Crude intermediates, CH_3_CN, 2,2-DMP (182.4 mL, 1.48 mol), and CSA (51.6 g, 0.222 mol), were added to the dried 5-L reaction flask, and the mixture was stirred at room temperature for 60 min. The reaction progress was monitored by TLC (ethyl acetate/hexane = 2/1 and ethyl acetate/hexane = 1/3). If the reaction was incomplete, the mixture was directly concentrated to dryness and step 2 was repeated with the addition of CH_3_CN and 2,2-DMP. Upon completion of the reaction, the mixture was quenched with Et_3_N, concentrated to dryness, and subjected to thin-layer chromatography using ethyl acetate/hexane = 1/2 as the eluent. The concentrated product was then subjected to azeotropic distillation with toluene (45 °C, below 25 mbar). The resulting solid was allowed to precipitate and washed thrice with hexane. The product was dried under vacuum to obtain **7** (169.2 g, 72% over two steps) as a yellow solid. The specific rotation was [α]^28^_D_ −196.2 (*c* 1.0, CHCl_3_). The IR spectrum (thin film) showed absorption bands at *ν* 3,447, 2,984, and 1,065 cm^–1^.

#### 2.2.9 *p*-Methylphenyl 2,3,4-tri-*O*-acetyl-*β*-D-xylopyranosyl-(1→4)-2,3-*O*-isopropylidene-1-thio-*α*-L-rhamnopyranoside (**15**)

Compound **12** (50 g, 0.181 mol), CCl_3_CN (500 mL), and Cs_2_CO_3_ (176.9 g, 0.543 mol) were sequentially added to the reaction flask and stirred at RT for approximately 30 min. The reaction progress was monitored by TLC (ethyl acetate/hexane = 1/2). The reaction mixture was filtered through Celite and concentrated to obtain crude product **6**. This crude product was then subjected to vacuum drying for 16 h together with compound **7** (56.2 g, 0.181 mol). Then, 5 Å molecular sieve powder was activated by heating and allowed to cool to room temperature. It was then added to the reaction flask along with CH_2_Cl_2_, and the mixture was stirred for 1 h. The reaction mixture was cooled to −78 °C, followed by the addition of 6.5 mL of TMSOTf. The mixture was stirred for 2 h. The temperature of the reaction mixture was gradually increased to 0 °C, and an additional 16.4 mL of TMSOTf was added. The mixture was stirred for 3 h. The reaction progress was monitored by TLC (ethyl acetate/hexane = 1/2). The reaction mixture was filtered through Celite, neutralized with Na_2_CO_3(aq)_, and extracted with CH_2_Cl_2_. The organic layer was dried over MgSO_4_, filtered, and concentrated. The residue was purified by column chromatography (silica gel; ethyl acetate/hexane = 2/3) to yield **15** (42.73 g, 53%). The specific rotation was [α]^28^_D_ −193.6 (*c* 1.0, CHCl_3_). The IR spectrum (thin film) showed absorption peaks at *ν* 2,984, 1,757, 1,230, and 1,054 cm^–1^. The ^1^H NMR spectrum (600 MHz, CDCl_3_) displayed signals at δ 7.36–7.30 (m, 2H, Ar-H), 7.10 (d, *J* = 7.9 Hz, 2H, Ar-H), 5.62 (s, 1H, H-1), 5.18 (t, *J* = 8.5 Hz, 1H, H-2′), 4.99 (d, *J* = 6.8 Hz, 1H, H-1′), 4.96–4.86 (m, 2H, H-4′, H-3′), 4.27 (d, *J* = 5.5 Hz, 1H, H-2), 4.13–4.02 (m, 3H, H-3, H-5, H-5a′), 3.57 (dd, *J* = 10.0, 7.5 Hz, 1H, H-4), 3.33 (dd, *J* = 11.8, 8.6 Hz, 1H, H-5b′), 2.31 (s, 3H, CH_3_), 2.08 (s, 3H, CH_3_), 2.02 (s, 6H, CH_3_), 1.49 (s, 3H, CH_3_), 1.33 (s, 3H, CH_3_), and 1.18 (d, *J* = 6.2 Hz, 3H, H-6). The ^13^C NMR spectrum (150 MHz, CDCl_3_) exhibited resonances at δ 170.1 (C), 169.9 (C), 169.7 (C), 138.0 (C), 132.7 (CH), 129.9 (CH), 129.3 (C), 109.6 (C), 99.4 (CH), 84.05 (CH), 79.0 (CH), 77.9 (CH), 77.0 (CH), 71.4 (CH), 71.0 (CH), 69.1 (CH), 65.56 (CH), 62.0 (CH_2_), 27.9 (CH_3_), 26.5 (CH_3_), 21.2 (CH_3_), 20.8 (CH_3_), 20.7 (CH_3_), 17.3 (CH_3_), and 14.2 (CH_3_). HRMS (ESI) analysis showed a peak at *m/z* 586.2319, which is consistent with the calculated value of *m/z* 586.2317 for C_27_H_36_O_11_SNH_4_ ([M + NH_4_]^+^).

#### 2.2.10 *p*-Methylphenyl 2,3,4-tri-*O*-acetyl-*β*-D-xylopyranosyl-(1→4)-2,3-di-*O*-acetyl-1-thio-α-L-rhamnopyranoside (**4**)

A solution of **15** (4.6 g, 8.1 mmol) in 2% HCl/MeOH (100 mL) was prepared and stirred for 16 h. The resulting mixture was evaporated and then azeotropically distilled with toluene (50 mL) twice under reduced pressure. After drying under high vacuum, the crude syrup was treated with Ac_2_O (2.2 mL, 23.1 mmol), Et_3_N (5.2 mL, 38.1 mmol), and DMAP (9 mg, 0.074 mmol) in CH_2_Cl_2_ under an N_2_ atmosphere at RT. Upon completion of the reaction after 2 h, the mixture was diluted with CH_2_Cl_2_, washed with H_2_O and brine, dried over MgSO_4_, and then concentrated under reduced pressure. The residue was purified by column chromatography (silica gel; ethyl acetate/hexane = 2/3) to yield **4** (3.8 g, 81%). The specific rotation was [α]^28^_D_ −132.9 (*c* 1.0, CHCl_3_). The IR spectrum (thin film) showed absorption bands at *ν* 2,940, 1,751, 1,223, and 1,054 cm^–1^. The ^1^H NMR spectrum (600 MHz, CDCl_3_) exhibited signals at δ 7.34–7.30 (m, 2H, Ar-H), 7.09 (d, *J* = 7.9 Hz, 2H, Ar-H), 5.37 (dd, *J* = 3.4, 1.6 Hz, 1H, H-2), 5.24 (d, *J* = 1.6 Hz, H-1), 5.20 (dd, *J* = 9.7, 3.4 Hz, 1H, 1H, H-3), 5.12 (t, *J* = 9.2 Hz, 1H, H-3′), 4.94 (td, *J* = 9.3, 5.4 Hz, 1H, H-4′), 4.88 (dd, *J* = 9.5, 7.6 Hz, 1H, H-2′), 4.64 (d, *J* = 7.6 Hz, 1H, H-1′), 4.22 (dq, *J* = 9.5, 6.2 Hz, 1H, H-5), 4.10 (dd, *J* = 11.7, 5.4 Hz, 1H, H-5′a), 3.70 (t, *J* = 9.6 Hz, 1H, H-4), 3.32 (dd, *J* = 11.8, 9.6 Hz, 1H, H-5′b), 2.29 (s, 3H, CH_3_), 2.08 (d, *J* = 13.0 Hz, 6H, CH_3_), 2.03–1.98 (m, 9H, CH_3_), and 1.29 (d, *J* = 6.2 Hz, 3H, CH_3_). The ^13^C NMR spectrum (150 MHz, CDCl_3_) displayed resonances at δ 170.3 (C), 169.9 (C), 169.9 (C), 169.6 (C), 169.6 (C), 138.2 (C), 132.7 (CH), 129.9 (CH), 129.3 (C), 101.07 (CH), 85.7 (CH), 76.5 (CH), 72.2 (CH), 71.7 (CH), 71.6 (CH), 71.1 (CH), 69.2 (CH), 68.1(CH), 62.5 (CH_2_), 21.1 (CH_3_), 21.0 (CH_3_), 20.9 (CH_3_), 20.8 (CH_3_), 20.7 (CH_3_), 20.5 (CH_3_), and 17.4 (CH_3_). HRMS (ESI) analysis showed a peak at *m/z* 630.2215, which is consistent with the calculated value of *m/z* 630.2215 for C_28_H_36_O_13_SNH_4_ ([M + NH_4_]^+^).

#### 2.2.11 Benzyl 2,3,4-tri-*O*-acetyl-*β*-D-xylopyranosyl-(1→4)-2,3-di-*O*-acetyl-*α*-L-rhamnopyranosyl-(1→2)-6-*O*-acetyl-3,4-*O*-isopropylidene-*β*-D-galactopyranoside (**16**)

To a stirred suspension of **4** (53.0 mg, 0.086 mmol), **5** (25.4 mg, 0.072 mmol), and activated 5 Å molecular sieve powder in anhydrous CH_2_Cl_2_ (1.6 mL), NIS (19.5 mg, 0.086 mmol) and TfOH (1.5 μL, 0.017 mmol) were added at 0 °C under an N_2_ atmosphere. Upon completion of the reaction after 30 min, the reaction was quenched by the addition of Et_3_N, saturated NaHCO_3_, and 10% Na_2_S_2_O_3_ aqueous solution. After warming and stirring at RT for 1 h, the reaction mixture was filtered, diluted with CH_2_Cl_2_, washed with 10% Na_2_S_2_O_3_ aqueous solution, saturated NaHCO_3_, and brine, dried over MgSO_4_, and concentrated under reduced pressure. The residue was purified by column chromatography (silica gel; ethyl acetate/hexane = 1/2) to yield **16** (46.1 mg, 76%) as a white solid. The specific rotation was [α]^26^_D_ −70.2 (*c* 1.5, CHCl_3_). The IR spectrum (thin film) showed absorption peaks at *ν* 2,935, 1,748, 1,223, and 1,051 cm^–1^. The ^1^H NMR spectrum (600 MHz, CDCl_3_) exhibited signals at δ 7.37–7.32 (m, 2H, Ar-H), 7.32–7.27 (m, 3H, Ar-H), 5.23–5.16 (m, 2H, H-2′, H-3′), 5.13–5.05 (m, 2H, H-1′, H-3′), 4.93 (td, *J* = 9.0, 5.3 Hz, 1H, H-4′), 4.89–4.80 (m, 2H, H-2″, Ar-CH_2_), 4.64–4.57 (m, 2H, H-1″, Ar-CH_2_), 4.39–4.26 (m, 3H, H-1, H-6a, H-6b), 4.15–4.10 (m, 1H, H-3), 4.07 (ddd, *J* = 11.5, 6.0, 3.5 Hz, 3H, H-4, H-5′, H-5a’’), 3.87 (ddd, *J* = 7.3, 4.7, 2.2 Hz, 1H, H-5), 3.74–3.68 (m, 1H, H-2), 3.58 (t, *J* = 9.6 Hz, 1H, H-4′), 3.31 (dd, *J* = 11.9, 9.2 Hz, 1H, H-5b’’), 2.10 (d, *J* = 7.8 Hz, 6H), 2.05 (s, 3H, CH_3_), 2.01 (s, 3H, CH_3_), 1.97 (d, *J* = 9.4 Hz, 6H, CH_3_), 1.47 (s, 3H, CH_3_), 1.27 (s, 3H, CH_3_), and 1.07 (d, *J* = 6.1 Hz, 3H, H-6′). The ^13^C NMR spectrum (150 MHz, CDCl_3_) displayed resonances at δ 170.79 (C), 170.25 (C), 170.03 (C), 169.88 (C), 169.86 (C), 169.28 (C), 136.45 (C), 128.57 (CH), 128.53 (CH), 128.05 (CH), 110.62 (C), 101.02 (CH), 98.72 (CH), 96.24 (CH), 79.72 (CH), 76.49 (CH), 75.80 (CH), 73.65 (CH), 72.15 (CH), 71.70 (CH), 71.14 (CH), 70.80 (CH), 70.48 (CH_2_), 70.01 (CH), 69.30 (CH), 66.82 (CH), 63.45 (CH_2_), 62.41 (CH_2_), 27.87 (CH_3_), 26.32 (CH_3_), 21.03 (CH_3_), 20.88 (CH_3_), 20.74 (CH_3_), 20.69 (CH_3_), 20.42 (CH_3_), and 17.20 (CH_3_). HRMS (ESI) analysis showed a peak at *m/z* 858.3399, which is consistent with the calculated value of *m/z* 858.3390 for C_39_H_52_O_20_NH_4_ ([M + NH_4_]^+^).

#### 2.2.12 Benzyl 2,3,4-tri-*O*-acetyl-*β*-D-xylopyranosyl-(1→4)-2,3-di-*O*-acetyl-*α*-L-rhamnopyranosyl-(1→2)-6-*O*-methanesulfonyl-3,4-*O*-isopropylidene-*β*-D-galactopyranoside (**17**)

To a stirred suspension of **16** (50.3 mg, 0.059 mmol) in anhydrous CH_2_Cl_2_ (5.0 mL), 7%–8% Mg(OMe)_2_ (0.5 mL) was added at 0 °C under an N_2_ atmosphere. Upon completion of the reaction after 1 h, the mixture was diluted with CH_2_Cl_2_, washed with H_2_O and brine, dried over MgSO_4_, and then concentrated under reduced pressure. The resulting mixture was evaporated and then azeotropically distilled with toluene (50 mL) twice under reduced pressure to generate crude intermediate **3**. After drying under high vacuum, the crude syrup containing **3** was treated with Et_3_N (16.6 μL, 0.119 mmol) and MsCl (9.2 μL, 0.119 mmol) at 0 °C in anhydrous CH_2_Cl_2_ (5 mL) under an N_2_ atmosphere. Upon completion of the reaction after 1 h, the mixture was diluted with CH_2_Cl_2_, washed with H_2_O and brine, dried over MgSO_4_, and then concentrated under reduced pressure. The residue was purified by column chromatography (silica gel; ethyl acetate/hexane = 2/3) to yield **17** (36.2 mg, 70% over two steps). The specific rotation was [α]^28^_D_ −38.2 (*c* 1.0, CHCl_3_). The IR spectrum (thin film) showed absorption bands at *ν* 2,935, 1,693, 1,295, and 1,193 cm^–1^. The ^1^H NMR spectrum (600 MHz, CDCl_3_) exhibited signals at δ 7.39–7.33 (m, 2H, Ar-H), 7.30 (td, *J* = 6.4, 1.7 Hz, 3H, Ar-H), 5.23–5.16 (m, 2H, H-2′, H-3′), 5.14–5.08 (m, 2H, H-1′, H-3″), 4.94 (td, *J* = 9.1, 5.4 Hz, 1H, H-4″), 4.89–4.82 (m, 2H, 2-H″, ArCH_2_), 4.64–4.60 (m, 2H, H-1′, ArCH_2_), 4.49–4.39 (m, 2H, H-6a, H-6b), 4.34 (d, *J* = 8.1 Hz, 1H, H-1), 4.17 (dd, *J* = 6.9, 5.6 Hz, 1H, H-3), 4.10–4.03 (m, 3H, H-4, H-5′, H-5a), 4.00 (ddd, *J* = 7.7, 4.4, 2.2 Hz, 1H, H-5), 3.71 (dd, *J* = 8.2, 6.9 Hz, 1H, H-2), 3.59 (t, *J* = 9.6 Hz, 1H, H-4′), 3.31 (dd, *J* = 11.8, 9.3 Hz, 1H, H-5b’’), 3.04 (s, 3H, SO_2_CH_3_), 2.11 (s, 3H, CH_3_), 2.06 (s, 3H, CH_3_), 2.02 (s, 3H, CH_3_), 1.98 (s, 3H, CH_3_), 1.97 (s, 3H, CH_3_), 1.47 (s, 3H, CH_3_), 1.27 (s, 3H, CH_3_), and 1.09 (d, *J* = 6.2 Hz, 3H, H-6′). The ^13^C NMR spectrum (150 MHz, CDCl_3_) displayed resonances at δ 170.2 (C), 169.9 (C), 169.8 (C), 169.7 (C), 169.3 (C), 136.2 (C), 128.6 (CH), 128.5 (CH), 128.1 (CH), 110.8 (C), 101.0 (CH), 98.9 (CH), 96.3 (CH), 79.8 (CH), 76.4 (CH), 75.7 (CH), 73.1 (CH), 72.1 (CH), 71.6 (CH), 71.1 (CH), 70.8 (CH_2_), 70.7 (CH), 69.9 (CH), 69.3 (CH), 68.7 (CH_2_), 66.9 (CH), 62.4 (CH_2_), 37.4 (CH_3_), 27.8 (CH_3_), 26.3 (CH_3_), 20.9 (CH_3_),20.7 (CH_3_), 20.6 (CH_3_), 20.4 (CH_3_), and 17.2 (CH_3_). HRMS (ESI) analysis showed a peak at *m/z* 894.3065¸ which is consistent with the calculated value of *m/z* 894.3060 for C_38_H_52_O_21_SNH_4_ ([M + NH_4_]^+^).

#### 2.2.13 Benzyl 2,3,4-tri-*O*-acetyl-*β*-D-xylopyranosyl-(1→4)-2,3-di-*O*-acetyl-*α*-L-rhamnopyranosyl-(1→2)-6-deoxy-6-iodo-3,4-*O*-isopropylidene-*β*-D-galactopyranoside (**18**)

To a solution of **17** (10.2 mg, 0.012 mmol) in DMF (2 mL), tetrabutylammonium iodide (TBAI) (4.3 mg, 0.012 mmol) and KI (5.8 mg, 0.034 mmol) were added. The solution was stirred at 120 °C for 26 h, then cooled to room temperature, diluted with water, and extracted using ethyl acetate. The combined organic layer was washed with saturated aqueous Na_2_S_2_O_3_ and brine and then dried over anhydrous Na_2_SO_4_. The organic layer was concentrated *in vacuo*, and the residue was purified by silica gel chromatography (silica gel; ethyl acetate/hexane = 2/3) to yield **18** (6.3 mg, 58%). The specific rotation was [α]^27^_D_ 29.4 (*c* 0.25, CHCl_3_). The IR spectrum (thin film) showed absorptions bands at *ν* 3,401, 2,920, 1,749, 1,221, and 1,052 cm^–1^. The ^1^H NMR spectrum (600 MHz, CDCl_3_) exhibited signals at δ 7.33 (td, *J* = 13.9, 7.0 Hz, 5H, Ar-H), 5.20 (dd, *J* = 9.0, 2.6 Hz, 2H, H-2′, H-3), 5.14–5.06 (m, 2H, H-1′, H-3″), 4.97–4.85 (m, 3H, H-2″, H-4″, Ar-CH_2_), 4.68 (d, *J* = 11.7 Hz, 1H, Ar-CH_2_), 4.63 (d, *J* = 7.5 Hz, 1H, H-1″), 4.28 (d, *J* = 8.2 Hz, 1H, H-1), 4.21 (dd, *J* = 5.6, 2.3 Hz, 1H, H-3), 4.15–4.05 (m, 3H, H-4, H-5″, H-5a’’), 3.81 (dt, *J* = 10.1, 3.9 Hz, 1H, H-5), 3.71 (t, *J* = 7.5 Hz, 1H, H-2), 3.59 (t, *J* = 9.4 Hz, 1H, H-4′), 3.41 (td, *J* = 9.7, 9.2, 6.9 Hz, 2H, H-6a, H-6b), 3.32 (dd, *J* = 11.8, 9.2 Hz, 1H, H-5b’’), 2.11 (d, *J* = 1.8 Hz, 3H, CH_3_), 2.02 (d, *J* = 1.7 Hz, 3H, CH_3_), 1.98 (s, 3H, CH_3_), 1.97 (s, 3H, CH_3_), 1.47 (s, 3H, CH_3_), 1.29 (s, 3H, CH_3_), and 1.10 (d, *J* = 6.0 Hz, 3H, H-6′). The ^13^C NMR spectrum (150 MHz, CDCl_3_) displayed resonances at δ 170.2 (C), 170.0 (C), 169.7 (C), 169.2 (C), 136.4 (C), 128.6 (C), 128.5 (CH), 128.1 (CH), 110.4 (C), 101.0 (CH), 98.6 (CH), 96.1 (CH), 79.8, 75.5(CH), 74.2 (CH), 73.4 (CH), 72.1 (CH), 71.6 (CH), 71.1 (CH), 70.4 (CH_2_), 70.0 (CH), 69.3 (CH), 66.8 (CH), 62.4 (CH_2_), 27.8 (CH_3_), 26.3 (CH_2_), 21.0 (CH_2_), 20.7 (CH_3_), 20.7 (CH_3_), 20.4 (CH_3_), and 17.2 (CH_3_). HRMS (ESI) analysis showed a peak at *m/z* 926.2298, which is consistent with the calculated value of *m/z* 926.2302 for C_37_H_49_IO_18_NH_4_ ([M + NH_4_]^+^).

#### 2.2.14 Benzyl 2,3,4-tri-*O*-acetyl-*β*-D-xylopyranosyl-(1→4)-2,3-di-*O*-acetyl-*α*-L-rhamnopyranosyl-(1→2)-3,4-*O*-isopropylidene-*β*-D-fucopyranoside (**19**)

From **4** and **10**: A mixture of acceptor **10** (5.0 mg, 0.017 mmol, 1 equiv), donor **4** (17.7 mg, 1.7 equiv, 0.029 mmol), and freshly activated AW-500 MS (25 mg) in dry CH_2_Cl_2_ (1.5 mL) was stirred at room temperature for 1 h under an N_2_ atmosphere and then cooled to 0 °C. NIS (6.5 mg, 1.7 equiv, 0.029 mmol) and TfOH (0.3 μL, 0.2 equiv, 0.003 mmol) were added to the reaction mixture. After constant stirring at 0 °C for 2 more hours, the mixture was filtered through a pad of Celite, the solids were washed with CH_2_Cl_2_, and the filtrate was washed with a mixture of saturated NaHCO_3(aq)_ and 20% Na_2_S_2_O_3_ solution and then with water. The organic layer was dried over anhydrous MgSO_4_, filtered, and concentrated *in vacuo*. The residue was purified by flash column chromatography (silica gel; ethyl acetate/hexane = 2/3) to afford trisaccharide **19** (12.7 mg, 95%).

From **18**: To a solution of **18** (10.1 mg, 0.011 mmol) in a co-solvent of THF/MeOH (10/1, 2 mL), Pd(OH)_2_/C (20% wt, 20.2 mg) was added. The resulting suspension was stirred under H_2(g)_ at room temperature and atmospheric pressure for 6 h. The reaction mixture was filtered through Celite and concentrated under reduced pressure. The residue was purified by silica gel chromatography (silica gel; ethyl acetate/hexane = 2/3) to yield **19** (8.2 mg, 95%).

The specific rotation was [α]^28^_D_ +38.0 (*c* 0.1, CHCl_3_). The IR spectrum (thin film) showed absorption bands at *ν* 2,919, 1,750, 1,222, and 1,073 cm^–1^. The ^1^H NMR spectrum (600 MHz, CDCl_3_) exhibited signals at δ 7.34 (t, *J* = 7.0 Hz, 2H, Ar-H), 7.31–7.26 (m, 3H, Ar-H), 5.24–5.17 (m, 2H, H-2′, H-3′), 5.13–5.07 (m, 2H, H-1′, H-3″), 4.97–4.90 (m, 1H, H-4″), 4.90–4.83 (m, 2H, H-2″, Ar-CH_2_), 4.62 (dd, *J* = 7.4, 2.1 Hz, H-1″), 4.58 (dd, *J* = 11.7, 2.0 Hz, 1H, Ar-CH_2_), 4.29 (dd, *J* = 8.2, 1.8 Hz, 1H, H-1), 4.12–4.04 (m, 3H, H-3, H-5′, H-5a’’), 3.93 (dt, *J* = 4.6, 2.0 Hz, 1H, H-4), 3.77 (qd, *J* = 6.6, 3.5 Hz, 1H, H-5), 3.73–3.67 (m, 1H, H-2), 3.57 (td, *J* = 9.6, 2.1 Hz, 1H, H-4′), 3.31 (ddd, *J* = 11.5, 9.1, 2.0 Hz, 1H, H-5b’’), 2.11 (s, 3H, CH_3_), 2.05 (s, 3H, CH_3_), 2.02 (s, 3H, CH_3_), 1.97 (s, 3H, CH_3_), 1.96 (s, 3H, CH_3_), 1.49 (s, 3H, CH_3_), 1.40 (dd, *J* = 6.5, 1.8 Hz, 3H, H-6), 1.29 (s, 3H, CH_3_), and 1.05 (dd, *J* = 6.5, 2.2 Hz, 3H, H-6′). The ^13^C NMR spectrum (150 MHz, CDCl_3_) displayed resonances at δ 170.2 (C), 170.0 (C), 169.8 (C), 169.8 (C), 169.2(C), 136.8 (C), 128.6 (CH), 128.4 (CH), 127.9 (CH), 110.0 (C), 100.9 (CH), 98.8 (CH), 96.2 (CH), 79.9 (CH), 75.8 (CH), 72.1 (CH), 71.8 (CH), 71.1 (CH), 70.3 (CH_2_), 70.1 (CH), 69.3 (CH), 68.8 (CH), 66.7 (CH), 62.4 (CH_2_), 28.0 (CH_3_), 26.4 (CH_3_), 21.0 (CH_3_), 20.7 (CH_3_), 20.7 (CH_3_), 20.4 (CH_3_), 17.2 (CH_3_), and 16.6 (CH_3_). HRMS (ESI) analysis showed a peak at *m/z* 800.3337, which is consistent with the calculated value of *m/z* 800.3335 for C_37_H_50_O_18_NH_4_ ([M + NH_4_]^+^).

#### 2.2.15 2,3,4-tri-*O*-acetyl-*β*-D-xylopyranosyl-(1→4)-2,3-di-*O*-acetyl-*α*-L-rhamnopyranosyl-(1→2)-3,4-*O*-isopropylidene-*β*-D-fucopyranoside (**2**)

From **18:** To a solution of **18** (7.1 mg, 0.008 mmol) in THF/MeOH (1/1, 1 mL), Pd(OH)_2_/C (20% wt, 35.5 mg) was added. The resulting suspension was stirred under H_2(g)_ at room temperature and atmospheric pressure for 7 h. The reaction mixture was filtered through Celite and concentrated under reduced pressure. The residue was purified by silica gel chromatography (silica gel; ethyl acetate/hexane = 2/3) to yield **2** (4.8 mg, 89%, *α:β* = 1.1:1.0).

From **19:** To a solution of **19** (10.4 mg, 0.012 mmol) in THF/MeOH (1/1, 1 mL), Pd(OH)_2_/C (20% wt, 52.0 mg) was added. The resulting suspension was stirred under H_2(g)_ at room temperature and atmospheric pressure for 4 h. The reaction mixture was filtered through Celite and concentrated under reduced pressure. The residue was purified by silica gel chromatography (silica gel; ethyl acetate/hexane = 2/3) to afford **2** (7.4 mg, 89%, *α:β* = 1.5:1.0).

The IR spectrum (thin film) showed absorption bands at *ν* 2,919, 1,750, 1,222, and 1,073 cm^–1^. The ^1^H NMR spectrum (600 MHz, CDCl_3_) exhibited signals at δ 5.24 (ddd, *J* = 22.0, 3.8, 1.8 Hz, 2H), 5.21–5.15 (m, 3H), 5.11 (td, *J* = 9.1, 2.8 Hz, 2H), 5.06 (d, *J* = 2.1 Hz, 2H), 4.96–4.90 (m, 2H), 4.85 (dd, *J* = 9.5, 7.7 Hz, 2H), 4.61 (td, *J* = 7.1, 6.5, 2.7 Hz, 3H), 4.37 (d, *J* = 7.1 Hz, 1H), 4.31 (dd, *J* = 7.2, 5.7 Hz, 1H), 4.15 (t, *J* = 6.2 Hz, 1H), 4.10 (dd, *J* = 11.8, 5.4 Hz, 2H), 4.07–4.02 (m, 2H), 3.98 (dd, *J* = 5.8, 2.1 Hz, 1H), 3.82–3.75 (m, 2H), 3.62 (t, *J* = 9.6 Hz, 2H), 3.57 (t, *J* = 7.1 Hz, 1H), 3.36–3.29 (m, 2H), 3.26 (dd, *J* = 6.3, 1.2 Hz, 1H), 2.85 (d, *J* = 4.1 Hz, 1H), 2.13 (d, *J* = 1.6 Hz, 6H), 2.05 (d, *J* = 1.6 Hz, 6H), 2.01 (d, *J* = 1.5 Hz, 6H), 1.99 (d, *J* = 1.5 Hz, 6H), 1.98 (d, *J* = 4.6 Hz, 6H), 1.49 (d, *J* = 7.7 Hz, 6H), 1.38 (d, *J* = 6.5 Hz, 3H), 1.33 (d, *J* = 6.6 Hz, 3H), 1.31 (d, *J* = 2.9 Hz, 6H), and 1.28–1.26 (m, 6H). The ^13^C NMR spectrum (150 MHz, CDCl_3_) displayed resonances at δ 170.2 (C), 170 (C), 169.9 (C), 169.8 (C), 169.6 (C), 169.5 (C), 110.0 (C), 109.1 (C), 101.1 (CH),101.0 (CH), 97.5 (CH), 97.1 (CH), 96.1 (CH), 94.8 (CH), 91.6 (CH), 78.6 (CH), 76.3 (CH), 76.3 (CH), 75.9 (CH), 75.4 (CH), 75.3 (CH), 75.1 (CH), 72.2 (CH), 72.1 (CH), 71.4 (CH), 71.2 (CH), 71.1 (CH), 70.1 (CH), 70.0 (CH), 69.2 (CH), 68.7 (CH), 67.3 (CH), 67.1 (CH), 63.4 (CH), 62.5 (CH_2_), 62.4 (CH_2_), 28.0 (CH_3_), 27.9 (CH_3_), 26.2 (CH_3_), 26.1 (CH_3_), 21.0 (CH_3_), 20.9 (CH_3_), 20.7 (CH_3_), 20.6 (CH_3_), 20.5 (CH_3_), 17.6 (CH_3_), 17.5 (CH_3_), 16.6 (CH_3_), and 16.5 (CH_3_). HRMS (ESI) analysis showed a peak at *m/z* 710.2867, which was consistent with the calculated value of *m/z* 710.2866 for C_30_H_44_O_18_NH_4_ ([M + NH_4_]^+^).

## 3 Results and discussion

### 3.1 Retrosynthesis

**Scheme 1:**
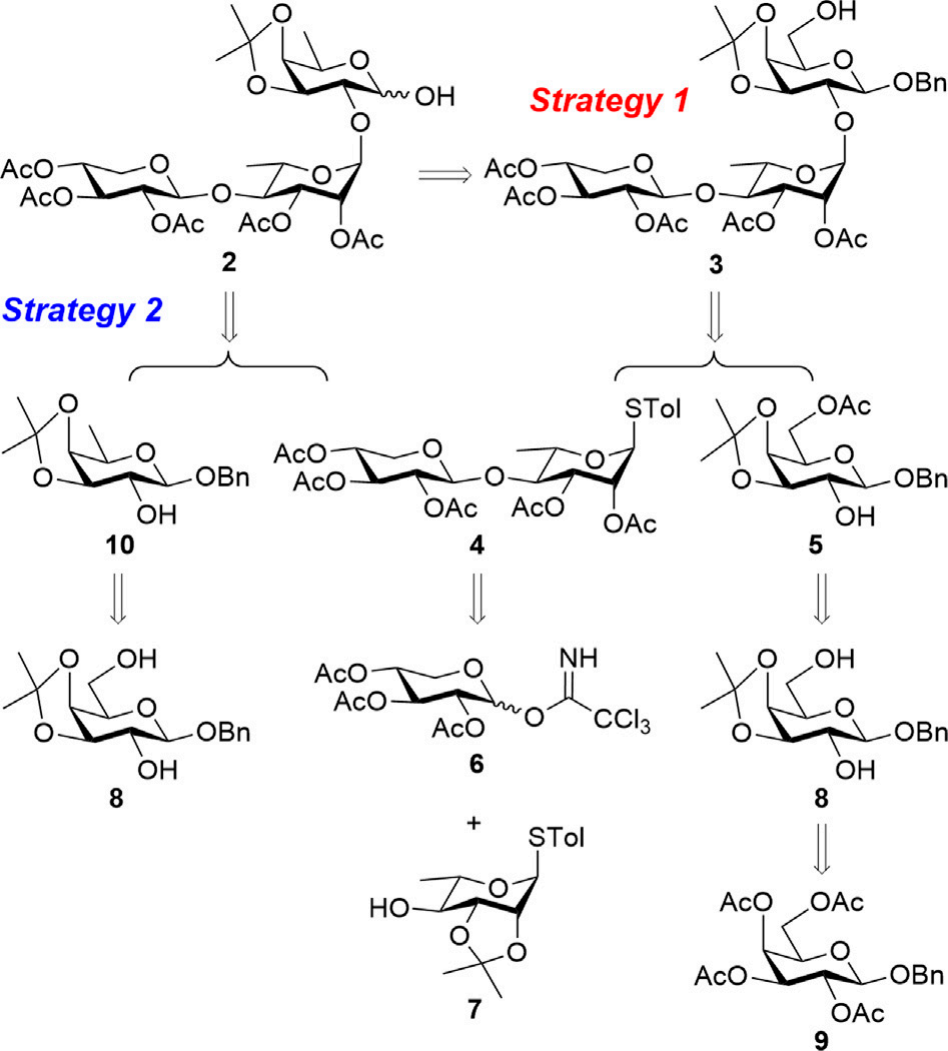
Structure of linear trisaccharide moiety **2** from QS-21 and its retrosynthesis. Ac, acetyl; Bn, benzyl; Tol, *p*-methylphenyl.

A linear trisaccharide of interest consists of D-xylose, L-rhamnose, and the rare D-fucose. Key challenges, such as the synthesis of the rare D-fucose unit and the stereocontrolled installation of 1,2-*trans* glycosidic bonds between each sugar ring, must be addressed to effectively generate the target linear trisaccharide. The target linear trisaccharide **2** can be synthesized using two [2 + 1] glycosylation strategies between a common disaccharide donor **4** and acceptors **5** and **10**, as shown in Scheme 1.

In the first retrosynthetic strategy, the target trisaccharide was envisioned to be obtained through a glycosylation reaction between glycosyl donor **4** and glycosyl acceptor **5**. The latter can be derived via regioselective acetylation of compound **8**, which, in turn, can be obtained through deacylation, followed by acetonide protection of compound **9**. After glycosylation, the D-galactose reducing end in **3** would be converted into a D-fucose moiety through deoxygenation at the C6 position. On the other hand, the second retrosynthetic strategy was to synthesize the target trisaccharide from donor **4** and acceptor **10**. Prior to glycosylation, D-galactose in **8** would be converted into a D-fucose acceptor **10**. Disaccharide donor **4**, used in both approaches, is synthesized via [1 + 1] glycosylation of D-xylose-derived donor **6** with L-rhamnose-derived acceptor **7**, ensuring efficient assembly. The glycosylation reactions are anticipated to yield 1,2-*trans*-glycosidic linkages facilitated by the adjacent acetyl (Ac) group at the C2 position.

### 3.2 Synthesis of the monosaccharide precursors

**Scheme 2:**
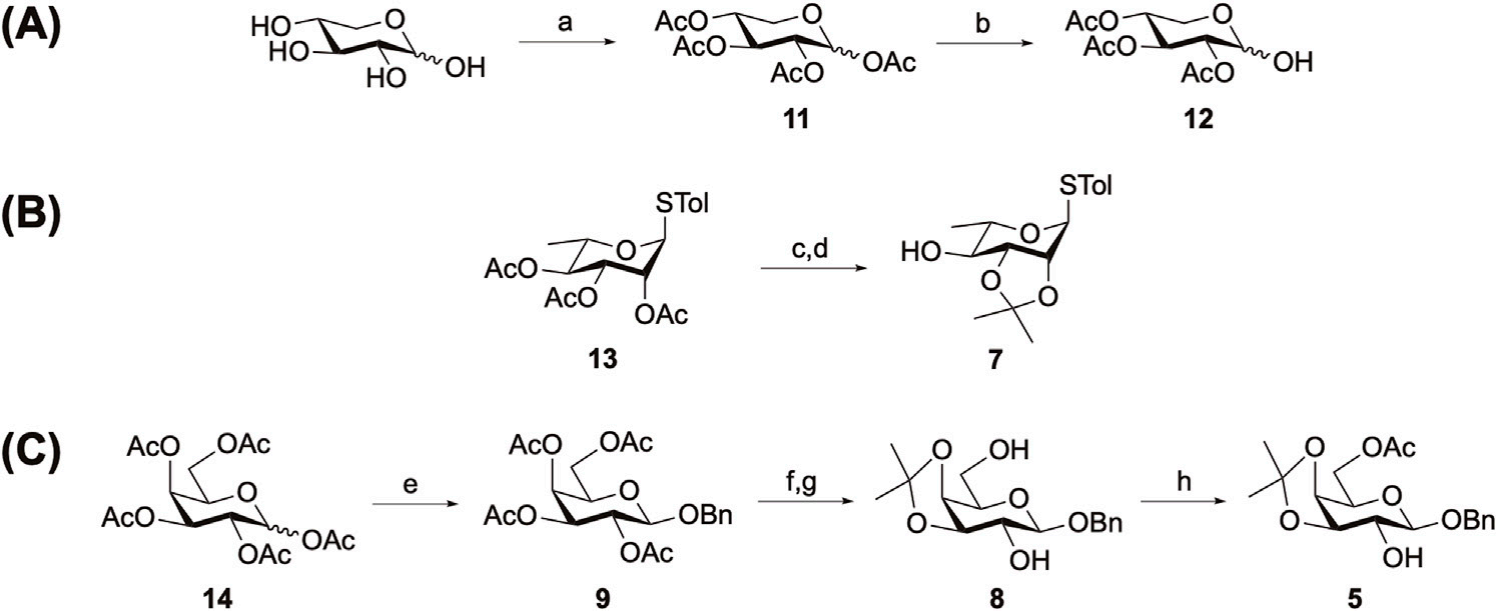
Synthesis of the monosaccharide building blocks **12 (A)**, **7 (B)**, and **5 (C)**. Reagents and conditions: (a) Ac_2_O, DMAP, Et_3_N, 0 °C, 3 h, 93%; (b) BnNH_2_, THF, 0 °C, 16 h, 68%; (c) NaOMe, MeOH, 0 °C, 1 h; (d) 2,2-DMP, CSA, MeCN, RT, 1 h, 72% over 2 steps; (e) BnOH, BF_3_•Et_2_O, 0 °C, 16 h, 75%; (f) NaOMe, MeOH, 0 °C to RT, 5 h; (g) 2,2-DMP, CSA, MeCN, RT, 30 min, 48% over two steps; (h) Ac_2_O, Et_3_N, CH_2_Cl_2_, 0 °C to RT, 3 h, 76%. DMAP, 4-dimethylaminopyridine; BnNH_2_, benzylamine; THF, tetrahydrofuran; Me, methyl; 2,2-DMP, 2,2-dimethoxypropane; CSA, 10-camphorsulfonic acid.

The truncated linear trisaccharide domain was synthesized beginning with the preparation of monosaccharide building blocks. In Scheme 2A, D-xylose building block **12** was synthesized starting from DMAP-catalyzed per-*O*-acetylation of D-xylose to generate **11** in 93% yield. Regioselective anomeric deacetylation with benzylamine (BnNH_2_) then afforded **12** in 68% yield. To prepare L-rhamnose-derived acceptor **7** (Scheme 2B), an acetonide protecting group was introduced via a sequential two-step process involving Zemplén deacylation, followed by isopropylidenation of the 2,3-*cis*-diol group using 2,2-DMP and CSA. This acetonide protection sequence resulted in the formation of **7** in 72% yield over two steps.

In Scheme 2C, per-*O*-acetylated D-galactose underwent BF_3_•Et_2_O-catalyzed benzyl group substitution at the anomeric position using benzyl alcohol (BnOH) to afford **9** in 75% yield. Subsequently, acetonide protection of **9** was performed analogously to the synthesis of **7**, resulting in the formation of **8** in 48% yield over two steps. The acetonide group was installed to protect the target trisaccharide prior to its final functionalization, when it was attached to the acyl side chain of QS-21. Finally, regioselective acetylation of the primary alcohol at the C6 position produced 2-alcohol **5** in 76% yield.

### 3.3 Synthesis of the common disaccharide donor

**Scheme 3:**
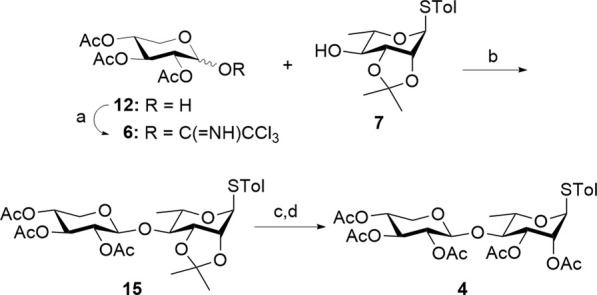
Synthesis of the common disaccharide donor **4**. Reagents and conditions: (a) Cl_3_CCN, Cs_2_CO_3_, RT, 0.5 h; (b) TMSOTf, AW-500, CH_2_Cl_2_, –78 °C to 0 °C, 5 h, 53%; (c) 2% HCl/MeOH, RT, 16 h; (d) Ac_2_O, DMAP, Et_3_N, EtOAc, 0 °C, 16 h, 81% over two steps. TMSOTf, trimethylsilyl trifluoromethanesulfonate; EtOAc, ethyl acetate.

The synthesis of common disaccharide donor **4** depicted in Scheme 3 involves the efficient assembly of compounds **6** and **7**. Conversion of **12** to a glycosyl trichloroacetimidate **6** using Cl_3_CCN was carried out, and then it was directly coupled with **7** through a TMSOTf-promoted glycosylation to afford disaccharide **15** in 53% yield. Neighboring group participation of the 2-*O*-Ac group from D-xylose resulted in a *β-*linked disaccharide (^1^ *J*_C–H_ = 162.9 Hz). Analysis of the nondecoupled HSQC spectra distinguished the *α*- and *β*-anomers, with the α-anomers displaying a J_C1–H1_ coupling of approximately 170 Hz for the anomeric carbon and proton, while the *β*-anomers exhibited around 160 Hz (Bock and Pedersen, 1974). Hydrolysis of the isopropylidene ketal in **15** using 2% HCl/MeOH, followed by acetylation, afforded common disaccharide donor **4** in 81% yield over two steps.

### 3.4 Synthesis of the truncated linear trisaccharide

**Scheme 4:**
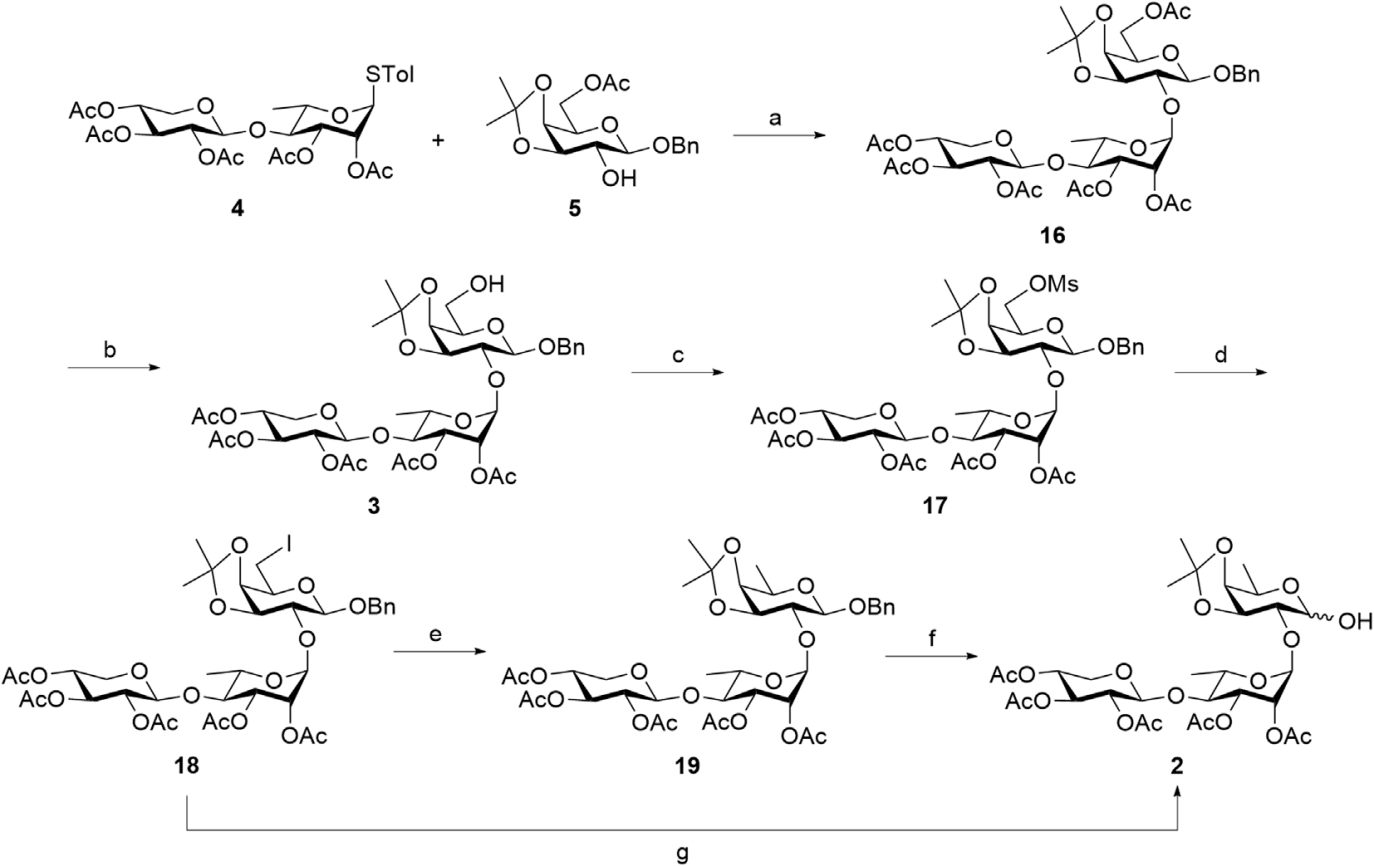
First approach in the synthesis of truncated linear trisaccharide domain **2**. Reagents and conditions: (a) NIS, TfOH, AW-500, CH_2_Cl_2_, 0 °C, 2 h, 76%; (b) 7%–8% Mg(OMe)_2_ in MeOH, CH_2_Cl_2_, 0 °C, 1 h; (c) Et_3_N, MsCl, CH_2_Cl_2_, 0 °C, 1 h, 70% (over two steps); (d) TBAI, KI, DMF, 120 °C, 22 h, 58%; (e) H_2(g)_, Pd(OH)_2_/C, THF/MeOH (10/1), RT, 6 h, 95%; (f) H_2(g)_, Pd(OH)_2_/C, THF/MeOH (1/1), RT, 4 h, 89%; (g) H_2(g)_, Pd(OH)_2_/C, THF/MeOH (10/1), RT, 7 h, 89%. NIS, *N*-iodosuccinimide; TfOH, trifluoromethanesulfonic acid; MsCl, methanesulfonyl chloride; TBAI, tetra-*n*-butylammonium iodide; DMF, *N*,*N*-dimethylformamide.

The synthesis of linear trisaccharide **2** was conducted using two distinct approaches. The first strategy, illustrated in Scheme 4, involved an NIS/TfOH-promoted [2 + 1] glycosylation between thioglycoside donor **4** and acceptor **5**. This reaction resulted in the successful formation of *α*-1→2-linked L-Rha-D-Gal **16** in 76% yield (^1^ *J*_C–H_ = 172.5 Hz), through the neighboring group participation of the 2-*O*-Ac moiety from the donor.

Upon obtaining D-galactose-containing trisaccharide **16**, post-glycosylation deoxygenation at the C6 position was performed to transform the reducing end into a rare D-fucose moiety. First, the primary 6-*O*-Ac group of D-galactose was selectively removed using 7%–8% Mg(OMe)_2_ in MeOH, generating 6-alcohol **3**, which was used directly in the next step without further purification. From this intermediate, deoxygenation was initially planned by introducing an iodide group at the C6 position, which could then be removed via hydrogenolysis (Lemieux and Levine, 1962). However, attempts to directly substitute the 6-hydroxy group in compound **3** with iodine on a 50 mg scale using the Mitsunobu reaction [PPh_3_, diisopropyl azodicarboxylate (DIAD), and methyl iodide] were unsuccessful. No product formation was observed, even after varying the solvent or increasing the reaction temperature. As shown in Table 1, the reaction in CH_2_Cl_2_ at 0 °C–22 °C (Entry 1) and 22 °C (Entry 2) failed to produce desired product **18**, and the starting material was largely recovered. Changing the solvent to THF and conducting the reaction at 0 °C–22 °C (Entry 3) or 60 °C (Entry 4) also did not lead to successful installation of a 6-iodo group at the reducing-end sugar. This may be attributed to the low reactivity of the hydroxy group toward direct iodine substitution.

**TABLE 1:** Direct substitution of the iodine atom through the Mitsunobu reaction.

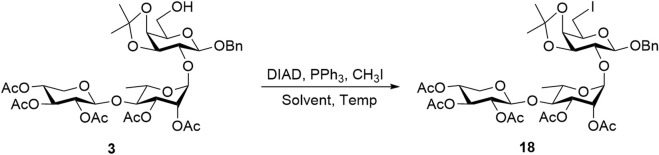				
Entry^a^	Solvent	Time	Temperature (°C)	Yield (%)
1	CH_2_Cl_2_	16 h	0–22	0
2	CH_2_Cl_2_	16 h	22	0
3	THF	16 h	0–22	0
4	THF	16 h	60	0

^a^ All reactions were carried out using 1.5 equiv DIAD, 3 equiv PPh_3_, and 1.5 equiv CH_3_I.

To overcome this key synthetic challenge, a methanesulfonyl (or mesyl) group was introduced at the O6 position prior to substitution with iodine to convert the hydroxy group to a more reactive leaving group. Following the deprotection of the 6-*O*-Ac group in trisaccharide **16** to form compound **3**, mesylation with mesyl chloride (MsCl) furnished **17** in 70% yield over two steps. Various reaction conditions were then explored to convert **17** into the C6-iodinated trisaccharide **18**, as summarized in Table 2. At a 10 mg scale, treatment of **17** with TBAI (1 equiv) and NaI (3 equiv) in DMF at 90 °C (Entry 1) did not yield **18**, even after 48 h, and the starting material remained unreacted. Increasing the temperature to 120 °C (Entry 2) also failed to produce the desired product. A trace amount of **18** was observed when KI (3 equiv) was used in place of NaI at 90 °C for 22 h (Entry 3). Gratifyingly, increasing the temperature to 120 °C under these conditions (Entry 4) successfully afforded the C6-iodinated trisaccharide **18** in 58% yield. This likely results from the higher solubility and enhanced nucleophilicity of iodide ions from KI in DMF at elevated temperature, facilitating more efficient substitution. The conversion from mesyl to iodide caused an upfield shift of the two H6 protons to 3.41 ppm and the disappearance of the mesyl CH_3_ proton signal at 3.04 ppm, suggesting the efficient formation of **18**. The final functionalization of trisaccharide **18** involves hydrogenolysis to remove the benzyl group and transform the galactosyl iodide moiety into a rare D-fucose residue. In our initial attempt, hydrogenolysis of compound **18** was carried out using Pd(OH)_2_/C (Degussa type, 20 wt%) at a loading rate of 1 g catalyst per gram of **18** under a hydrogen atmosphere. Under the initial conditions, using THF as the solvent, the reaction proceeded very slowly. Switching to an increased catalyst loading of 2 g Pd(OH)_2_ per gram of **18** and THF/MeOH (10/1) as co-solvent significantly improved the reaction efficiency, enabling selective removal of the iodine atom while preserving the benzyl group and affording compound **19** with a D-fucose-reducing end in 95% yield. The formation of D-fucose in **19** from the D-galactose-reducing end in **18** was evidenced by the presence of methyl protons at the C6 position (^1^H NMR: d, *δ* 1.44 ppm, *J* = 6.5 Hz). Furthermore, hydrogenolysis of **19** in the THF/MeOH (1/1) co-solvent using Pd(OH)_2_/C at a loading rate of 5 g catalyst per gram of starting material then furnished target linear trisaccharide **2** in 89% yield (*α*:*β* = 1:1).

**TABLE 2:** Substitution of the iodine atom with mesylated compound **17**.

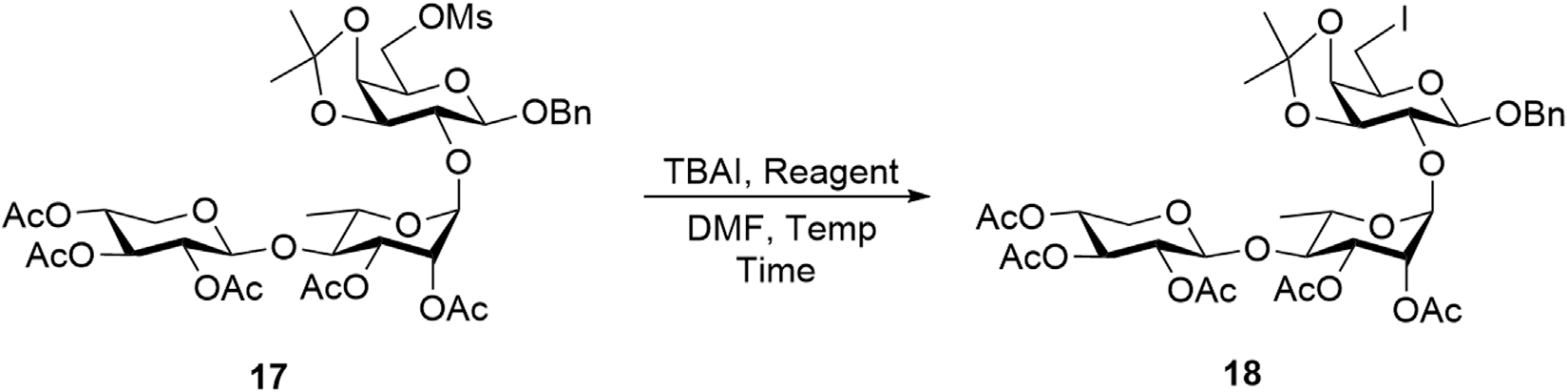				
Entry^a^	Reagent	Temperature (°C)	Time (h)	Yield (%)
1	NaI (3 equiv)	90	48	0
2	NaI (3 equiv)	120	48	0
3	KI (3 equiv)	90	22	Trace
4	KI (3 equiv)	120	22	58

^a^ All reactions were carried out using 1 equiv TBAI in 10 mg.

In our second attempt to carry out the final functionalization, we tried to synthesize trisaccharide **2** directly from **18** by investigating the effect of Pd(OH)_2_/C loading on the hydrogenolysis reaction. Hydrogenolysis of C–O bonds, such as those in benzyl protecting groups, typically requires harsher conditions than the cleavage of C–I bonds (Ahluwalia, 2023). Hydrogenolysis was performed using Pd(OH)_2_/C at a loading rate of 5 g catalyst per gram of **18** under an H_2_ atmosphere, with THF/MeOH (1/1) as the co-solvent. This condition effectively removed both the iodine atom and the benzyl protecting group from **18**, directly producing trisaccharide **2** in 89% yield. The successful synthesis of final compound **2** from **18** was confirmed by the disappearance of benzyl proton-NMR peaks and the detection of methyl protons at the C6 position (^1^H NMR: d, *δ* 1.38 ppm, *J* = 6.5 Hz).

Our second strategy in the synthesis of the truncated linear trisaccharide domain of QS-21 utilizes a pre-glycosylation deoxygenation approach from Scheme 1. Conversion of the D-galactose unit into rare D-fucose was conducted prior to [2 + 1] glycosylation with D-xylose and L-rhamnose-derived donor **4**. This strategy was anticipated to be more efficient than the initial approach due to its reduced number of steps since the 6-deoxygenation reaction is only conducted on a monosaccharide.

**Scheme 5:**
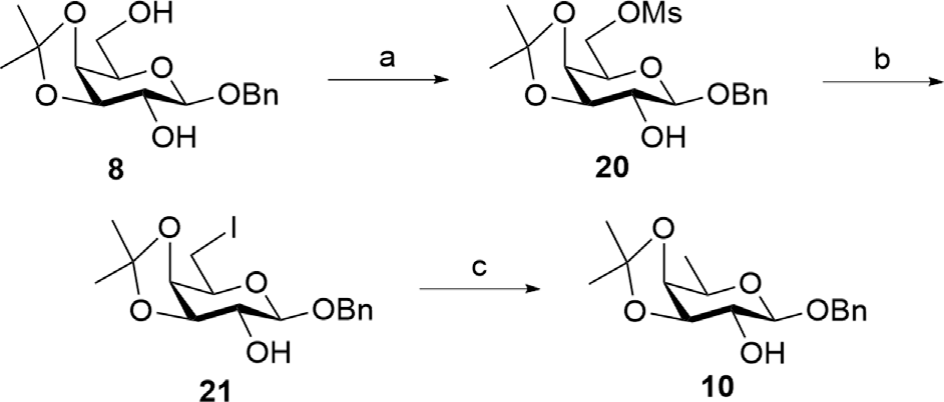
Synthesis of acceptor **10** through pre-glycosylation deoxygenation. Reagents and conditions: (a) Et_3_N, MsCl, CH_2_Cl_2_, 0 °C, 2 h, 63%; (b) TBAI, KI, DMF, 120 °C, 22 h, 72%; (c) H_2(g)_, Pd(OH)_2_/C, THF/MeOH (10/1), RT, 7 h, 95%.

The pre-glycosylation deoxygenation strategy commenced with the transformation of isopropylidenated D-galactose **8** into D-fucose through a series of mesylation, iodine substitution, and selective hydrogenolysis reactions (Scheme 5). Mesylation of 2,6-diol **8** at the C6 position furnished **20** in 63% yield, as indicated by the presence of mesyl methyl protons at 3.04 ppm. Following the optimized conditions from Table 2, the substitution of the OMs group with an iodine atom using TBAI and KI in DMF afforded **21** in 72% yield. Finally, D-fucose acceptor **10** was obtained following selective hydrogenolysis at the C6 position in THF/MeOH (10/1), preserving the integrity of the benzyl group prior to the [2 + 1] glycosylation step. The reaction was carried out using 1 g of Pd(OH)_2_/C per gram of **21** to achieve a high yield of 95% for compound **10**, mirroring the synthesis of trisaccharide **19**. The detection of an upfield chemical shift for the methyl C6-protons at 1.43 ppm of **10** indicates the successful conversion of D-galactose into rare D-fucose.

**Scheme 6:**
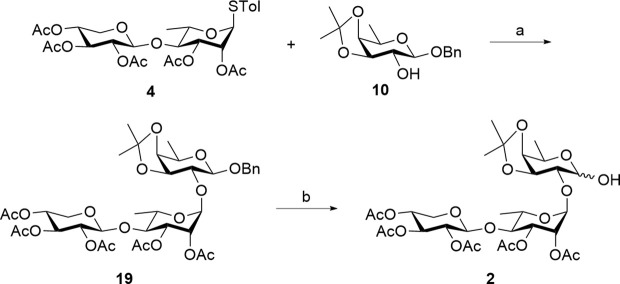
[2 + 1] glycosylation approach for the synthesis of truncated linear trisaccharide **2** using the pre-glycosylation deoxygenation strategy. Reagents and conditions: (a) NIS, TfOH, AW-500, CH_2_Cl_2_, 0 °C, 2 h, 95%; (b) H_2(g)_, Pd(OH)_2_/C, THF/MeOH (1/1), RT, 7 h, 89%.

With synthesized D-fucose acceptor **10** and disaccharide donor **4** in hand, we proceeded with [2 + 1] glycosylation to generate the trisaccharide unit **19** (Scheme 6). Using NIS/TfOH in CH_2_Cl_2_ at 0 °C, glycosylation proceeded with exclusive *α*-stereoselectivity (^1^ *J*_C–H_ = 173.2 Hz) to furnish trisaccharide **19** in 95% yield. Neighboring group participation of the 2-*O*-Ac group led to *α*-selectivity. Afterward, benzyl group deprotection through hydrogenolysis with H_2_ and Pd(OH)_2_/C in THF/MeOH (1/1) produced target linear trisaccharide **2** in 89% yield (*α*:*β* = 1.5:1.0). The structures of the final compound were confirmed through NMR spectroscopic and mass spectrometric analyses (see Supplementary Material).

**FIGURE 2:**
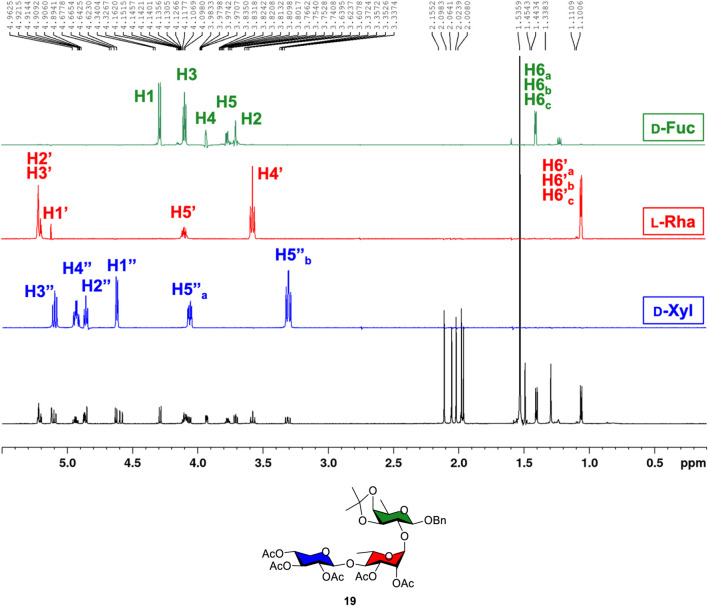
1D TOCSY analysis of trisaccharide **19** resolving the complex ^1^H NMR spectrum into three resolve spectra corresponding to the ring protons of D-xylose (blue), L-rhamnose (red), and D-fucose (green).

Using trisaccharide **19** as a representative example, all glycan backbone ^1^H protons were examined through the ^1^H, ^13^C, COSY, HSQC, HMBC, and 1D total correlation spectroscopy (1D TOCSY) experiments in detail. Following the identification of anomeric carbons and protons using HSQC, the complex ^1^H NMR spectrum was then deconvoluted into three distinct spectroscopic patterns by selectively exciting anomeric ^1^H nuclei at a given frequency using 1D TOCSY NMR. Figure 2 depicts the identification of protons from D-xylose (blue spectrum), L-rhamnose (red spectrum), and D-fucose (green spectrum) through a combined 1D TOCSY NMR and 2D COSY analysis. This combined analysis, along with HSQC and HMBC, confirms the connectivity of each sugar ring from trisaccharide **19**, as shown in Figure 2.

Comparing the post-glycosylation and pre-glycosylation deoxygenation strategies, target trisaccharide **2** was obtained in overall yields of 7.8% and 15.6%, respectively. The pre-glycosylation deoxygenation route required only a single-step transformation after the [2 + 1] glycosylation reaction to reach the final product, whereas the post-glycosylation pathway involved three additional steps. Although the pre-glycosylation approach includes a three-step synthesis of the deoxygenated D-fucose acceptor, this added effort is offset by the reduced number of steps following glycosylation. Overall, the higher yield and greater step economy highlight the pre-glycosylation deoxygenation strategy as the more efficient route to the truncated linear trisaccharide fragment of QS-21.

## 4 Conclusion

We have successfully accomplished an efficient synthesis of the truncated linear trisaccharide domain **2** of QS-21. Two synthetic strategies were used: post-glycosylation and pre-glycosylation deoxygenation. The latter proved to be more efficient, yielding a higher overall yield with fewer reaction steps. En route, we established an efficient synthesis of the rare D-fucose moiety from D-galactose. We believe that this efficient synthesis of the linear trisaccharide domain will be applicable to the synthesis of pure and homogeneous QS-21 structures and their analogs for use as vaccine adjuvants.

## Data Availability

The original contributions presented in the study are included in the article/Supplementary Material, further inquiries can be directed to the corresponding author.

## References

[B1] Ahluwalia V. K. (2023). “Hydrogenolysis,” in Reduction in organic synthesis, 115–121. 10.1007/978-3-031-37686-3_4

[B19] Bock K. Pedersen C. A. (1974). Study of 13C–H Coupling Constants in Hexopyranoses. J. Chem. Soc. Perkin Trans. 2 (293), 293–297. 10.1039/P29740000293

[B2] Chea E. K. Fernández-Tejada A. Damani P. Adams M. M. Gardner J. R. Livingston P. O. (2012). Synthesis and preclinical evaluation of QS-21 variants leading to simplified vaccine adjuvants and mechanistic probes. J. Am. Chem. Soc. 134, 13448–13457. 10.1021/ja305121q 22866694 PMC3436428

[B3] Fernández-Tejada A. Chea E. K. George C. Pillarsetty N. Gardner J. R. Livingston P. O. (2014). Development of a minimal saponin vaccine adjuvant based on QS-21. Nat. Chem. 6, 635–643. 10.1038/nchem.1963 24950335 PMC4215704

[B4] Fernández-Tejada A. Tan D. S. Gin D. Y. (2016). Development of improved vaccine adjuvants based on the saponin natural product QS-21 through chemical synthesis. Acc. Chem. Res. 49, 1741–1756. 10.1021/acs.accounts.6b00242 27568877 PMC5032057

[B5] Garçon N. Van Mechelen M. (2011). Recent clinical experience with vaccines using MPL- and QS-21-containing adjuvant systems. Expert Rev. Vaccines 10, 471–486. 10.1586/erv.11.29 21506645

[B6] Gin Y. Slovin F. (2011). Enhancing immunogenicity of cancer vaccines: QS-21 as an immune adjuvant. Curr. Drug. Ther. 6, 207–212. 10.2174/157488511796391988 25473385 PMC4248601

[B7] Kensil C. R. Patel U. Lennick M. Marciani D. (1991). Separation and characterization of saponins with adjuvant activity from *Quillaja saponaria* Molina cortex. J. Immunol. 146, 431–437. 10.4049/jimmunol.146.2.431 1987271

[B8] Lacaille-Dubois M.-A. (2019). Updated insights into the mechanism of action and clinical profile of the immunoadjuvant QS-21: a review. Phytomedicine 60, 152905. 10.1016/j.phymed.2019.152905 31182297 PMC7127804

[B9] Lemieux R. U. Levine S. (1962). The products of the prevost reaction on D-glucal triacetate. Can. J. Chem. 40, 1926–1932. 10.1139/v62-296

[B10] Liang P.-H. Lai Y.-H. Chang C.-K. Chaw C.-W. (2020). Saponin conjugate and vaccine or pharmaceutical composition comprising the same.

[B11] Marciani D. J. Pathak A. K. Reynolds R. C. Seitz L. May R. D. (2001). Altered immunomodulating and toxicological properties of degraded *Quillaja saponaria* Molina saponins. Int. Immunopharmacol. 1, 813–818. 10.1016/S1567-5769(01)00016-9 11357894

[B12] Martin L. B. B. Kikuchi S. Rejzek M. Owen C. Reed J. Orme A. (2024). Complete biosynthesis of the potent vaccine adjuvant QS-21. Nat. Chem. Bio. 20, 493–502. 10.1038/s41589-023-01538-5 38278997 PMC10972754

[B13] Pink J. R. Kieny M.-P. (2004). 4th Meeting on novel adjuvants currently in/close to human clinical testing. Vaccine 22, 2097–2102. 10.1016/j.vaccine.2004.01.021 15212010

[B14] Ragupathi G. Damani P. Deng K. Adams M. M. Hang J. George C. (2010). Preclinical evaluation of the synthetic adjuvant SQS-21 and its constituent isomeric saponins. Vaccine 28, 4260–4267. 10.1016/j.vaccine.2010.04.034 20450868 PMC2882175

[B15] Ragupathi G. Gardner J. R. Livingston P. O. Gin D. Y. (2011). Natural and synthetic saponin adjuvant QS-21 for vaccines against cancer. Expert Rev. Vaccines 10, 463–470. 10.1586/erv.11.18 21506644 PMC3658151

[B16] Reed S. G. Orr M. T. Fox C. B. (2013). Key roles of adjuvants in modern vaccines. Nat. Med. 19, 1597–1608. 10.1038/nm.3409 24309663

[B17] Reed J. Orme A. El-Demerdash A. Owen C. Martin L. B. B. Misra R. C. (2023). Elucidation of the pathway for biosynthesis of saponin adjuvants from the soapbark tree. Science 379, 1252–1264. 10.1126/science.adf3727 36952412

[B18] Wang P. Kim Y.-J. Navarro-Villalobos M. Rohde B. D. Gin D. Y. (2005). Synthesis of the potent immunostimulatory adjuvant QS-21A. J. Am. Chem. Soc. 127, 3256–3257. 10.1021/ja0422007 15755124

