# Supplementary material for: A streamlined synthetic approach to the truncated linear trisaccharide fragment of QS-21
Source: Front Chem. 2025 Sep 23;13:1650302. doi: 10.3389/fchem.2025.1650302 (PMC12502733; doi:10.3389/fchem.2025.1650302)
Supplement: Supplementary file 1 [file DataSheet1.pdf]

Supplementary Material

A Streamlined Synthetic Approach to the Truncated Linear Trisaccharide Fragment of QS-21

Jhe-Sian Lin[1,2], Zheng-Hao Tzeng[2], Jasper S. Dumalaog[2,3], and Shang-Cheng Hung[2,3,4,5]*

[1]Institute of Biochemistry and Molecular Biology, National Yang Ming Chiao Tung University, Taipei 112304, Taiwan

[2]Genomics Research Center, Academia Sinica, Taipei 11529, Taiwan

[3]Department of Chemistry, National Tsing Hua University, Hsinchu 30013, Taiwan

[4]Department of Applied Science, National Taitung University, Taitung 95092, Taiwan

[5]Department of Chemistry, National Cheng Kung University, Tainan 70101, Taiwan

*** Correspondence:**

Shang-Cheng Hung, schung@gate.sinica.edu.tw

1 Supplementary Figures (NMR Spectra of Synthesized Compounds)

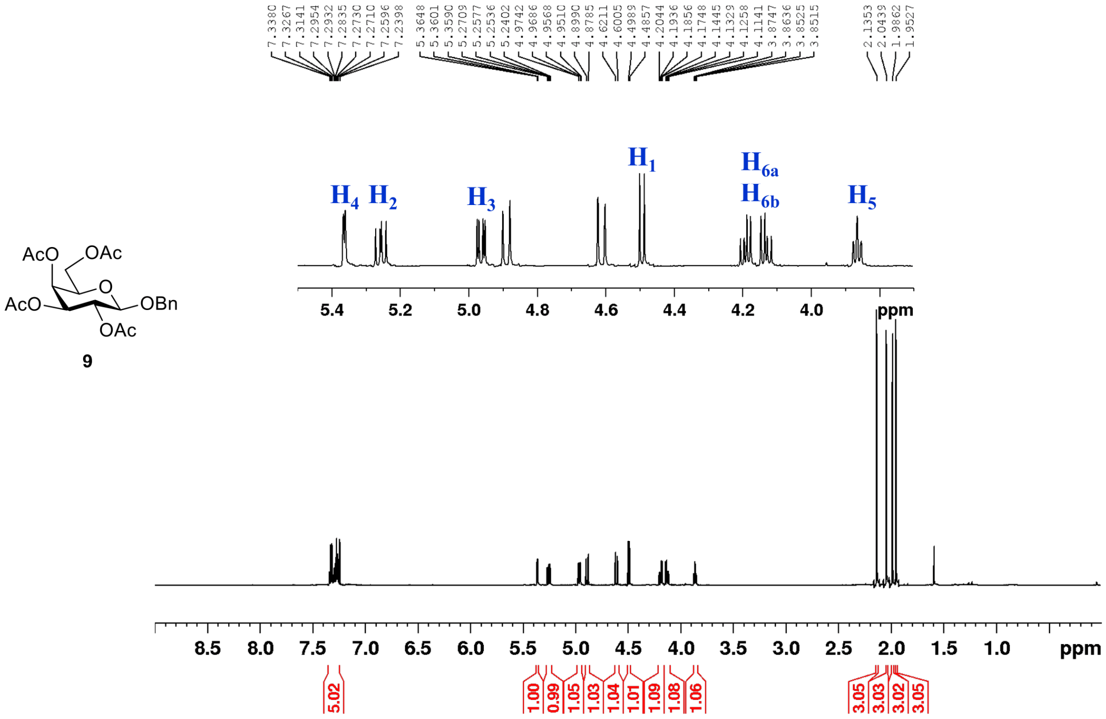

Supplementary Figure S1. ^{1}H NMR Spectrum of Compound **9**.

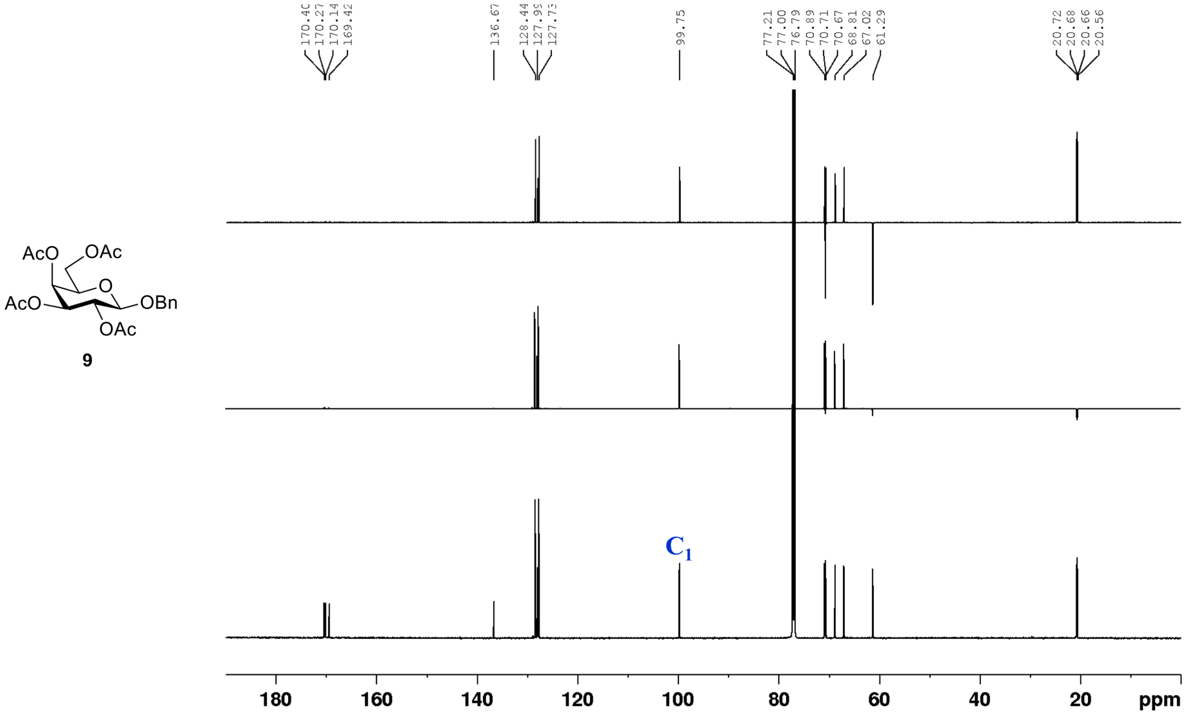

Supplementary Figure S2. ^{13}C NMR Spectrum of Compound **9**.

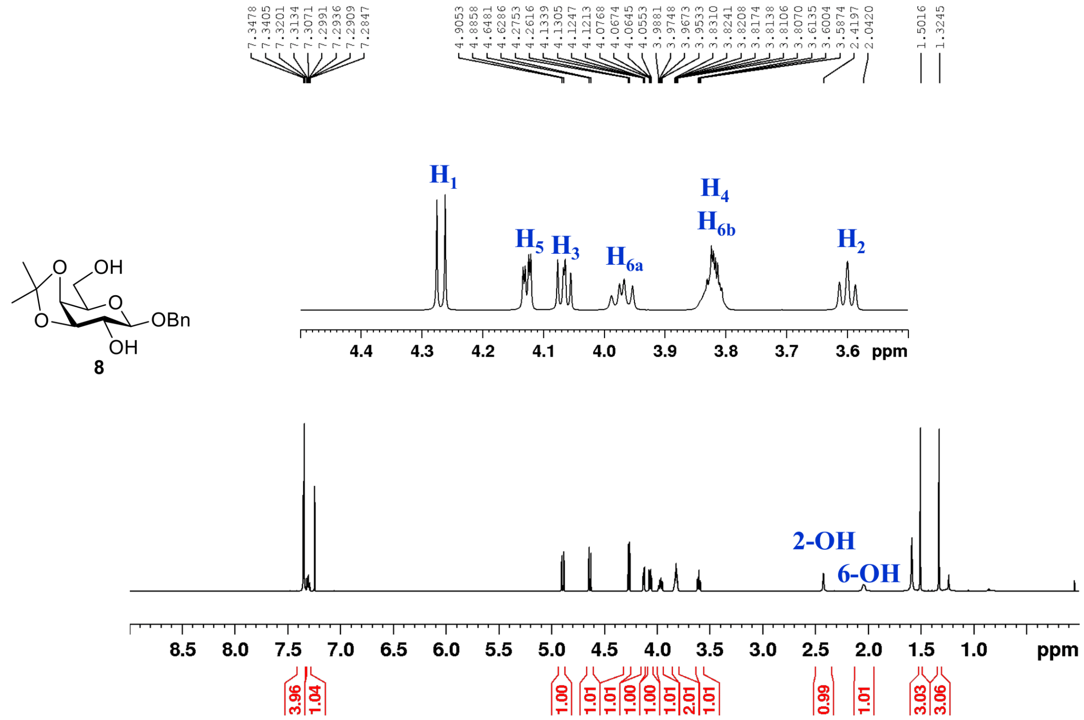

Supplementary Figure S3. ^{1}H NMR Spectrum of Compound **8**.

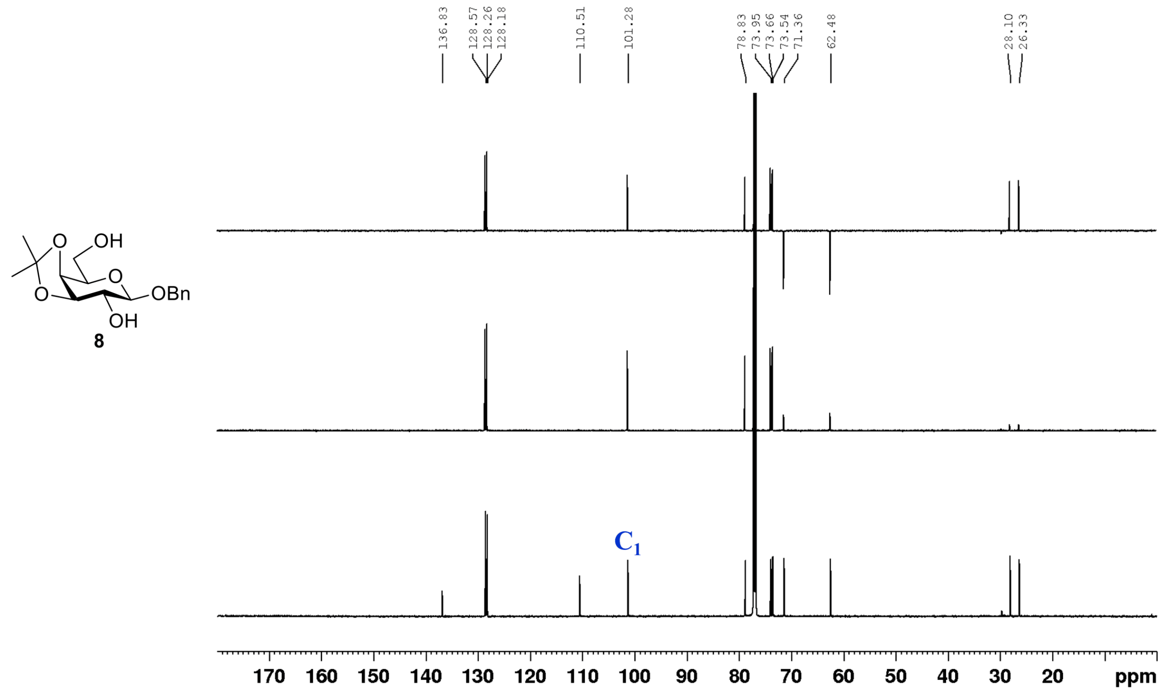

Supplementary Figure S4. ^{13}C NMR Spectrum of Compound **8**.

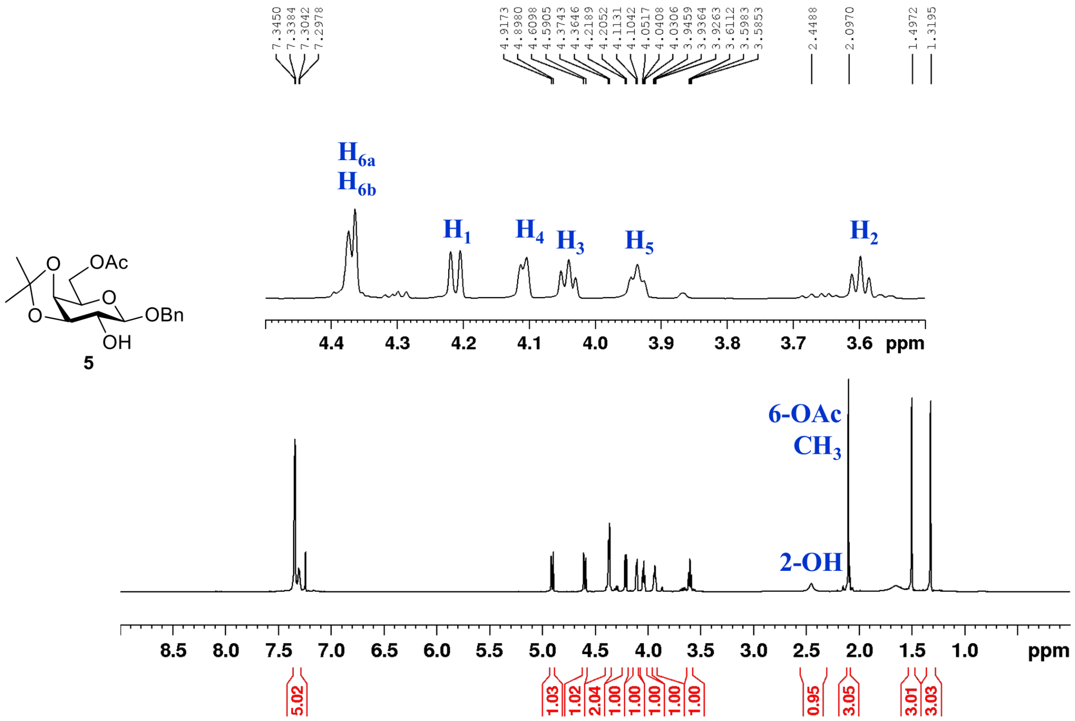

Supplementary Figure S5. ^{1}H NMR Spectrum of Compound **5**.

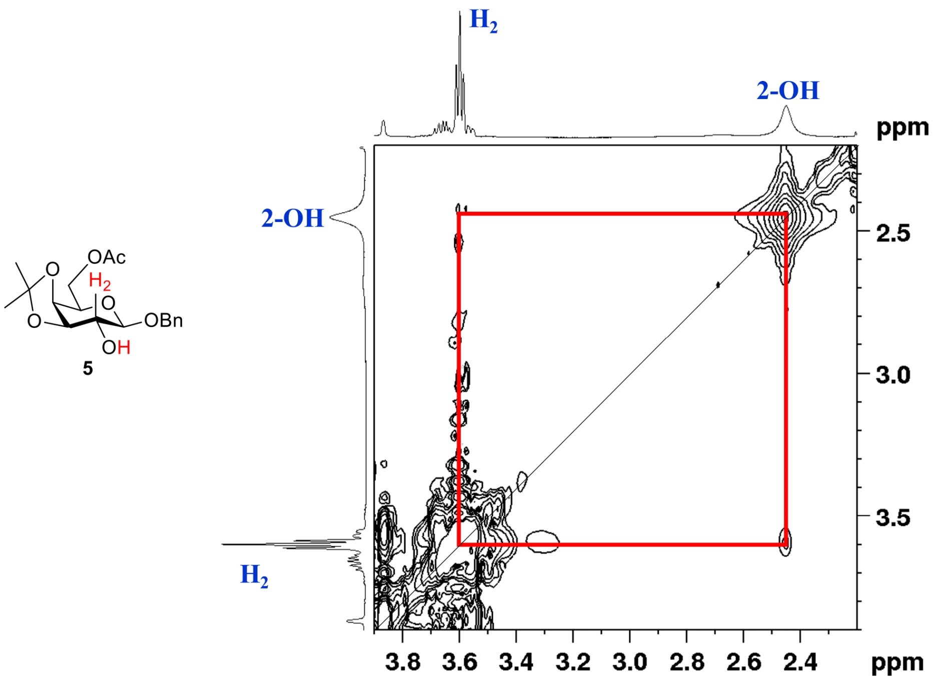

Supplementary Figure S6. 2D-COSY NMR Spectrum of Compound **5**.

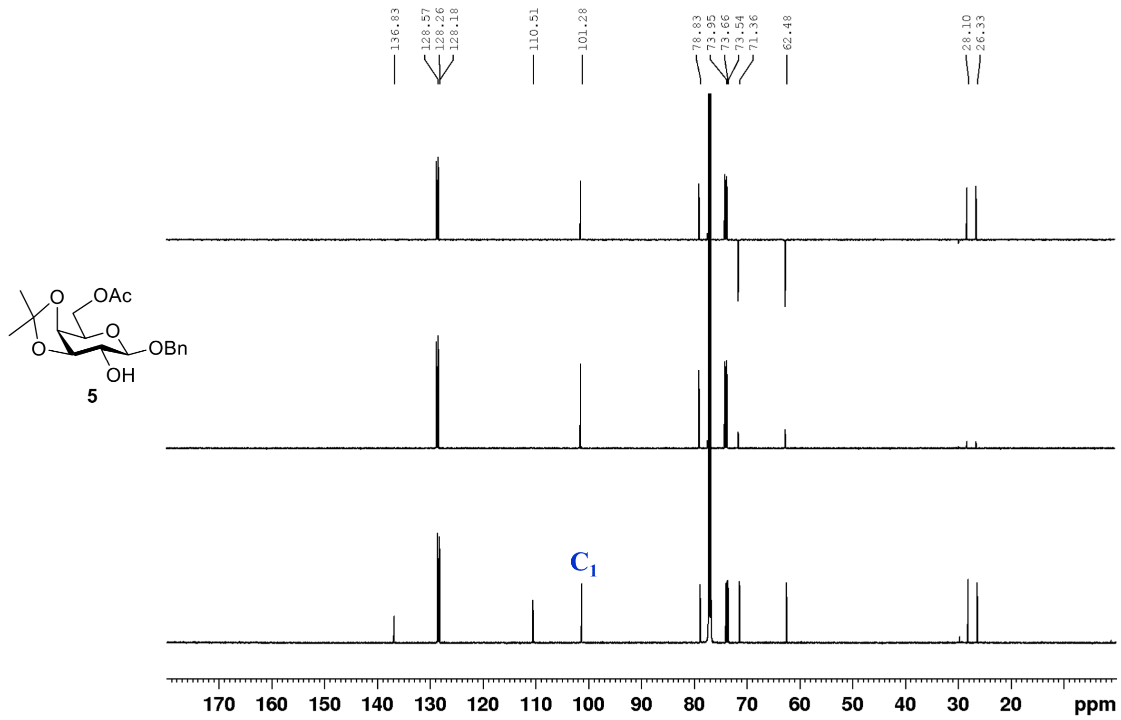

Supplementary Figure S7. ^{13}C NMR Spectrum of Compound **5**.

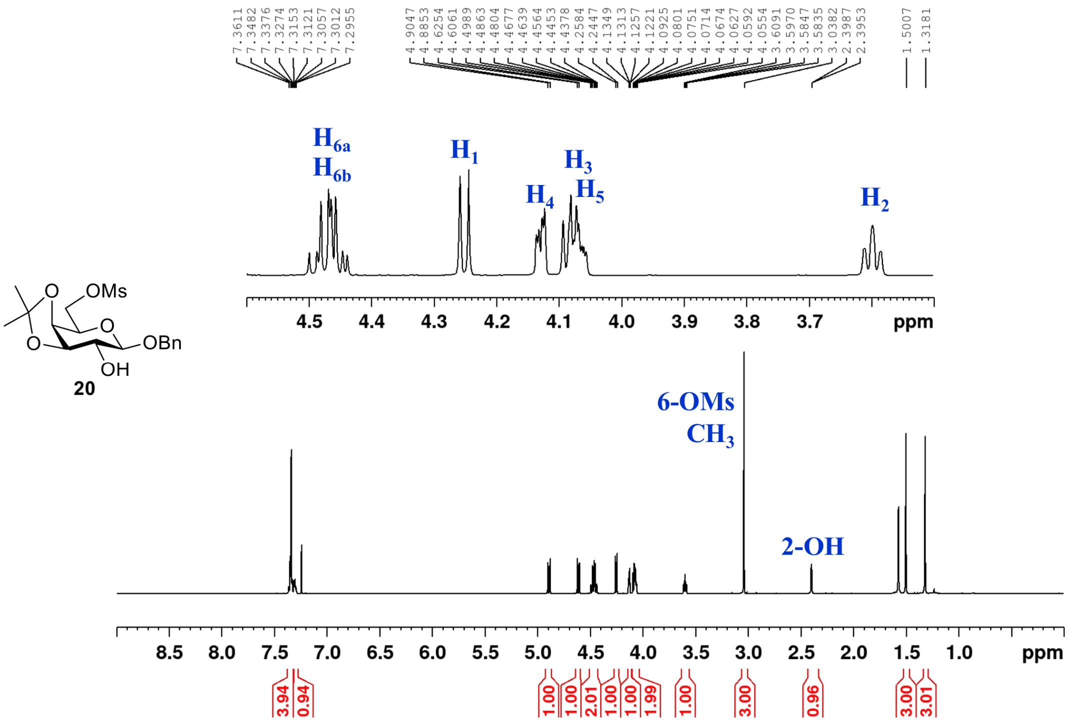

Supplementary Figure S8. ^{1}H NMR Spectrum of Compound **20**.

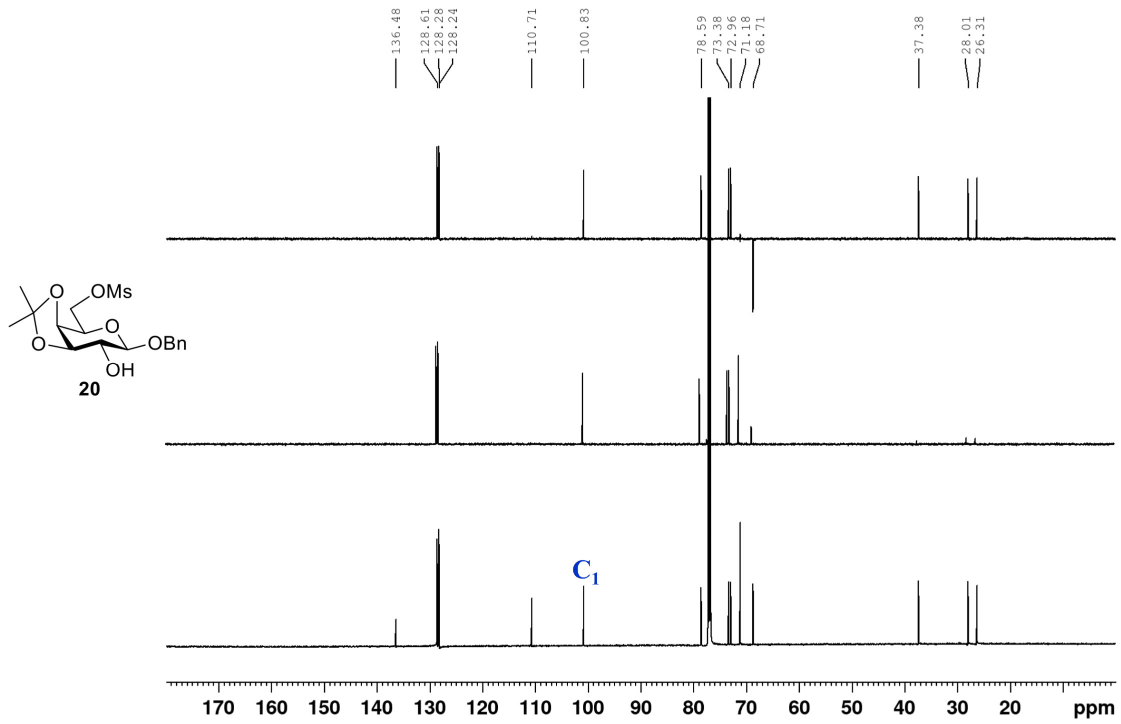

Supplementary Figure S9. ^{13}C NMR Spectrum of Compound **20**.

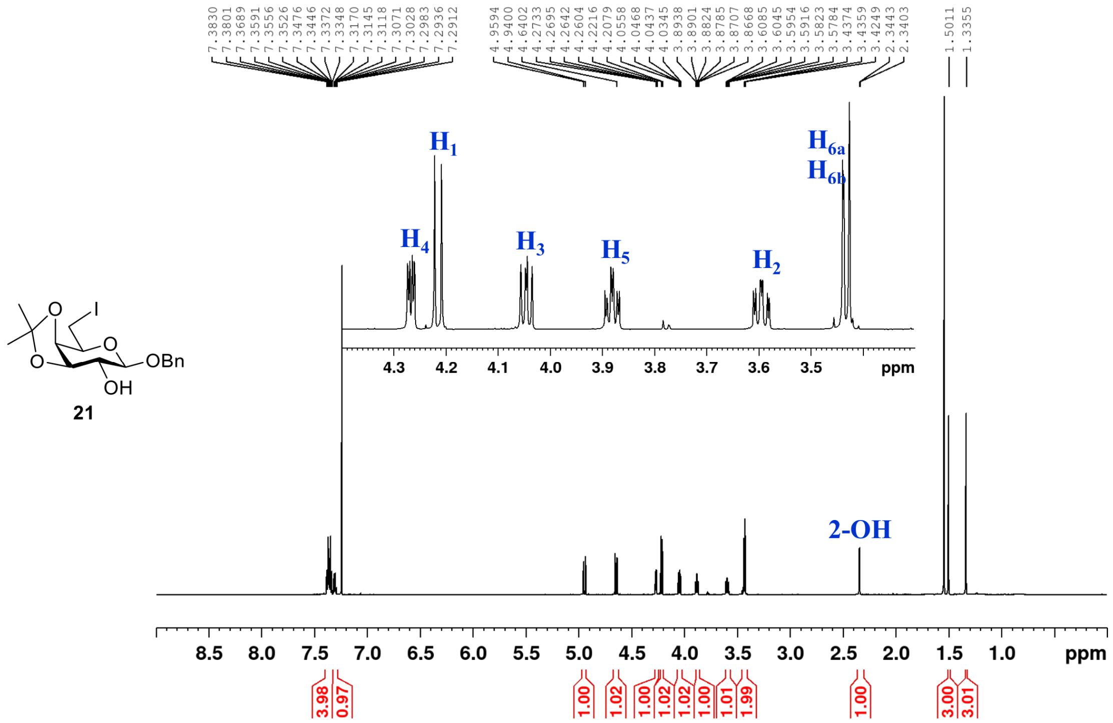

Supplementary Figure S10. ^{1}H NMR Spectrum of Compound **21**.

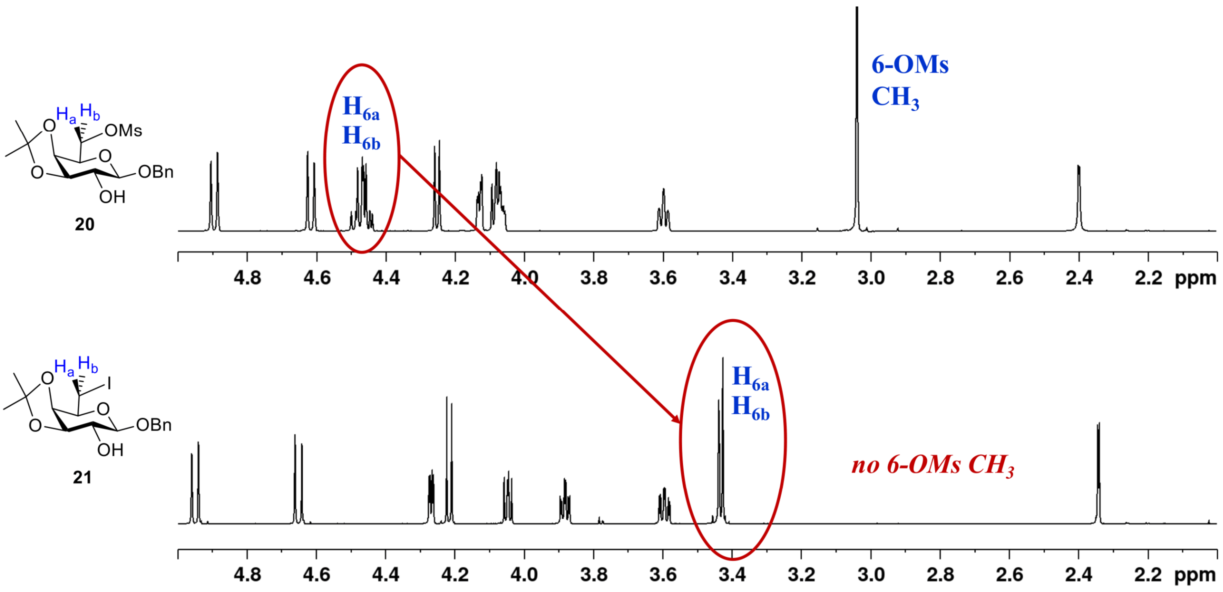

Supplementary Figure S11. Overlay ^{1}H NMR Spectra of **20** (top) and **21** (bottom).

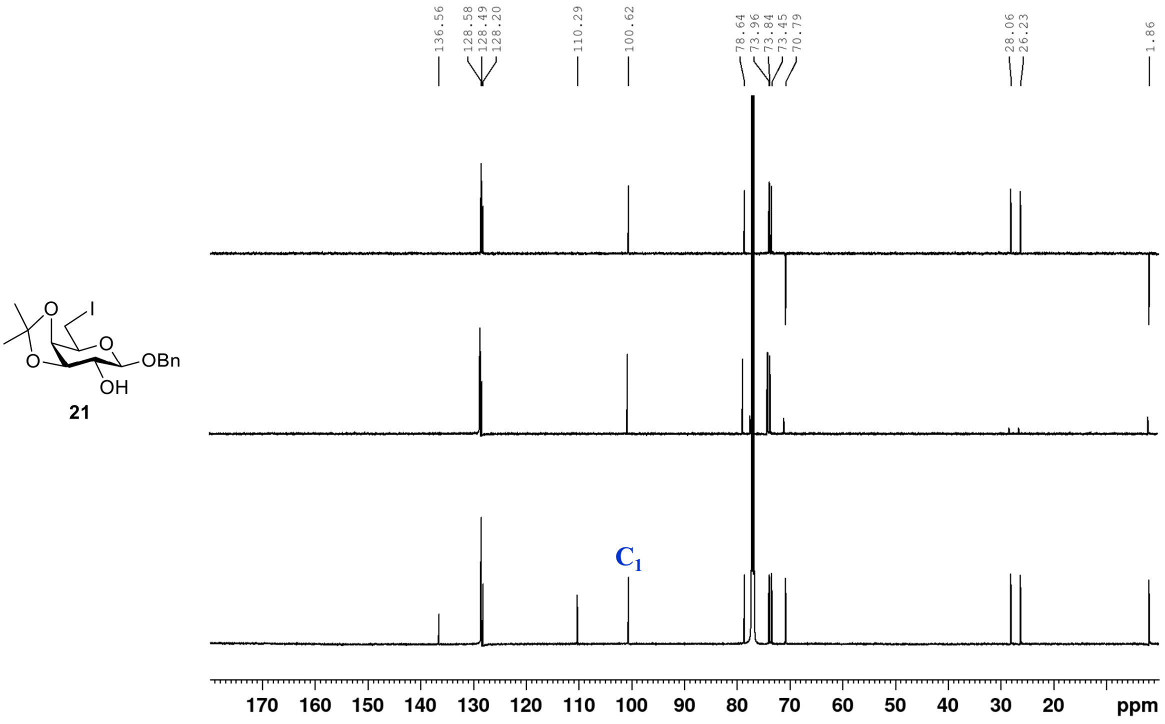

Supplementary Figure S12. ^{13}C NMR Spectrum of Compound **21**.

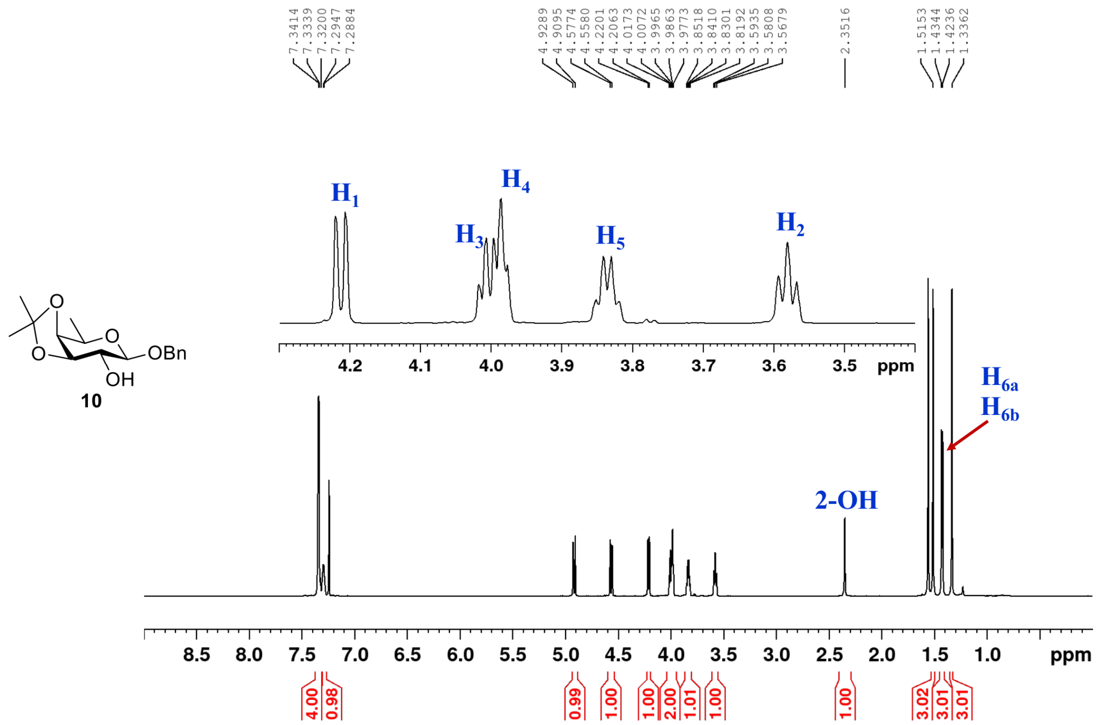

Supplementary Figure S13. ^{1}H NMR Spectrum of Compound **10**.

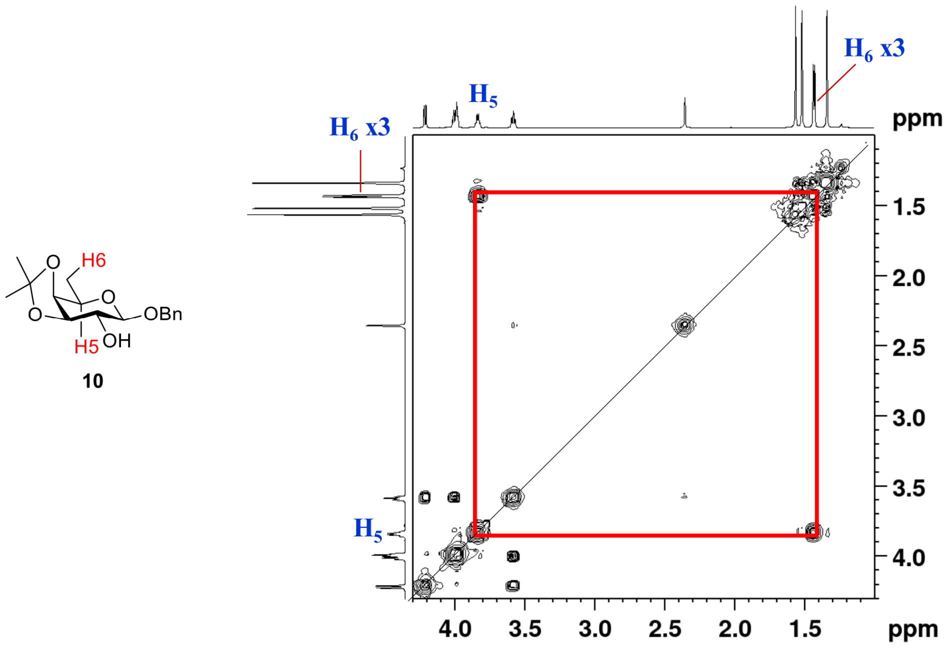

Supplementary Figure S14. 2D-COSY NMR Spectrum of Compound **10**.

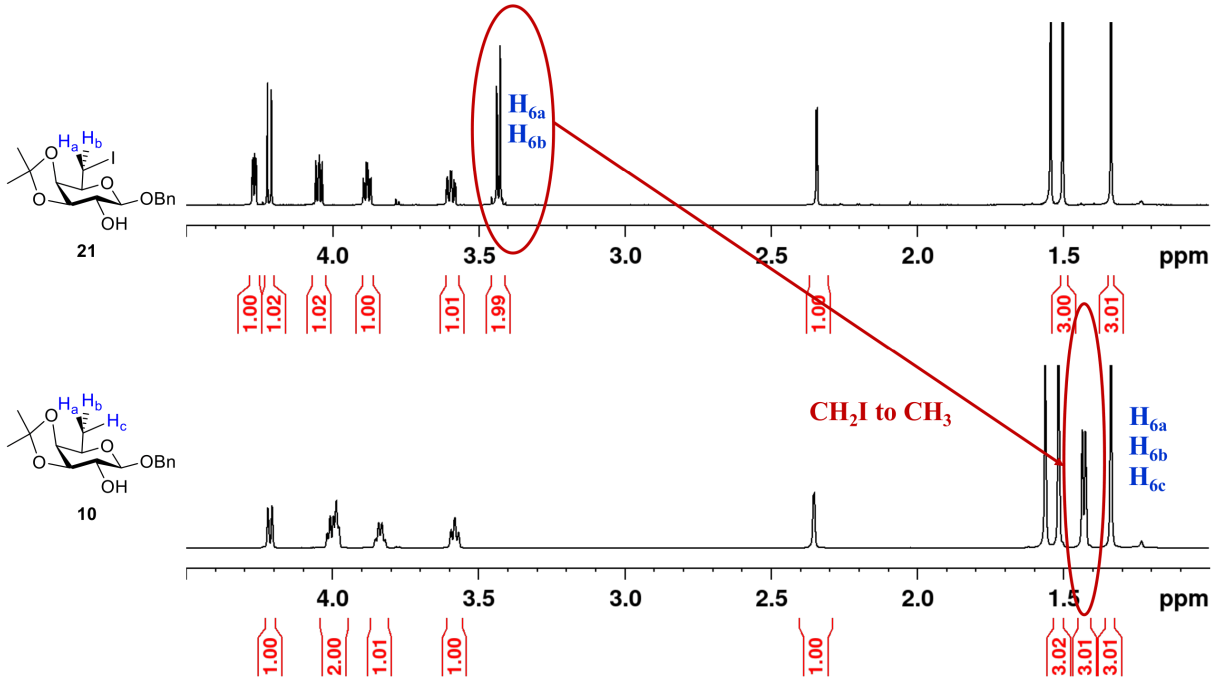

Supplementary Figure S15. Overlay ^{1}H NMR Spectra of **21** (top) and **10** (bottom).

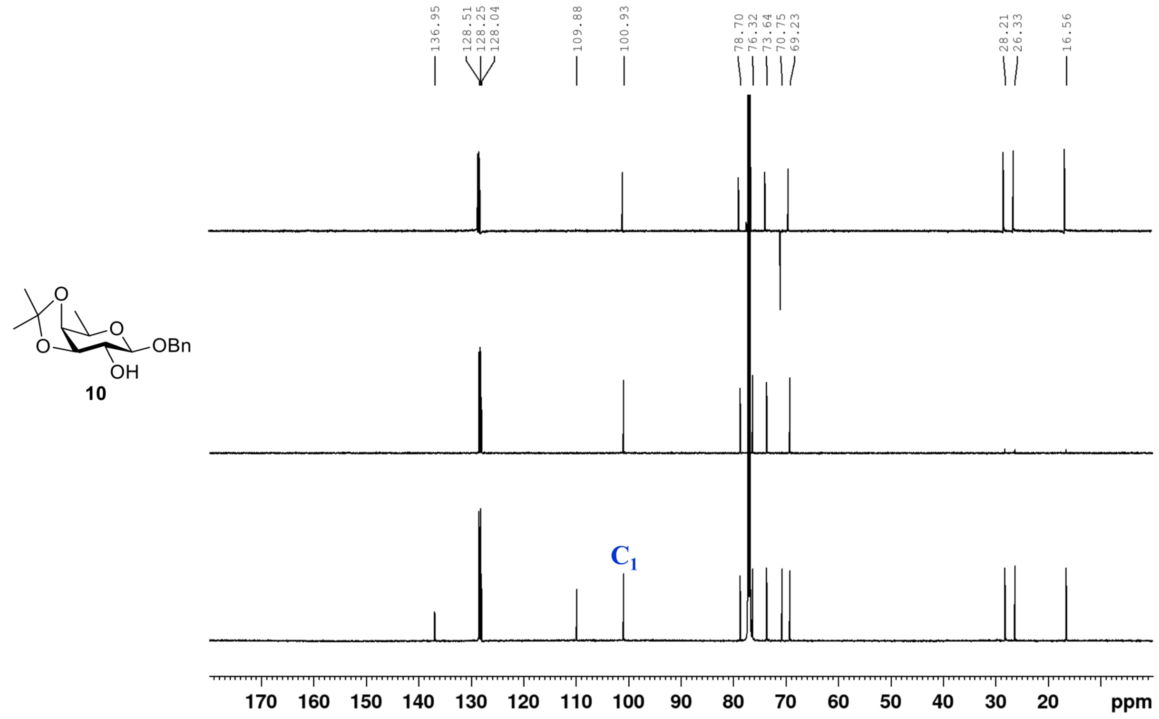

Supplementary Figure S16. ^{13}C NMR Spectrum of Compound **10**.

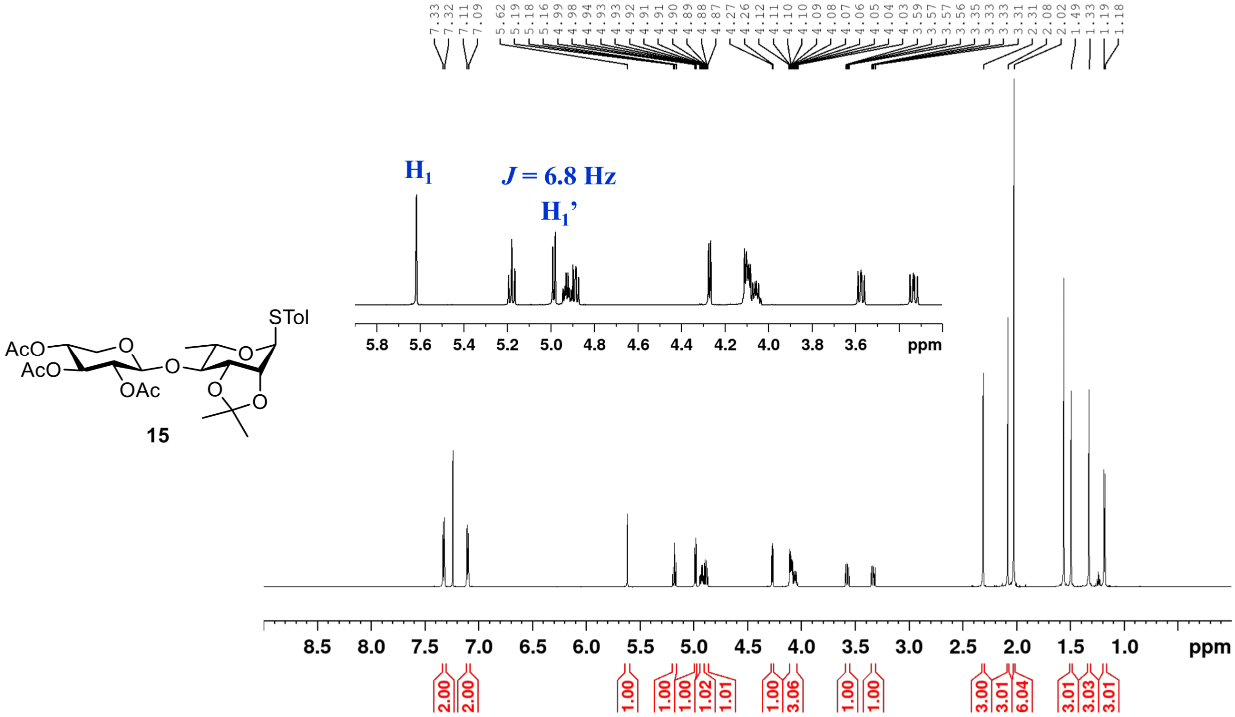

Supplementary Figure S17. ^{1}H NMR Spectrum of Compound **15**.

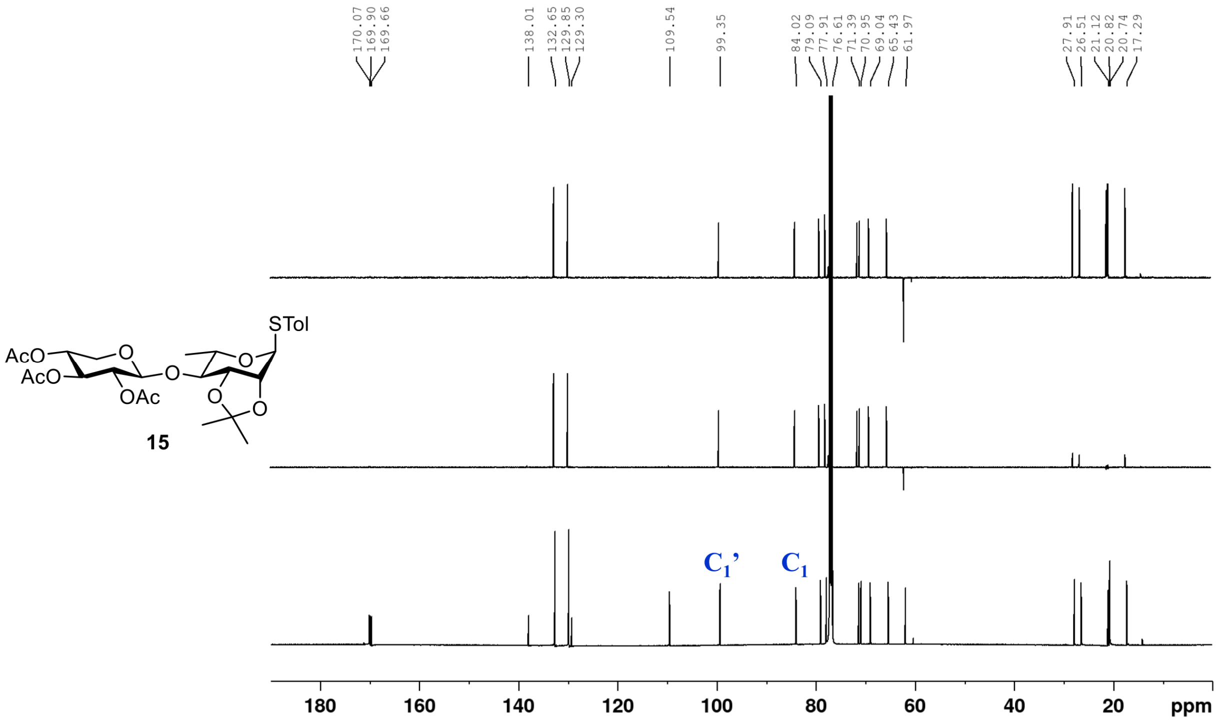

Supplementary Figure S18. ^{13}C NMR Spectrum of Compound **15**.

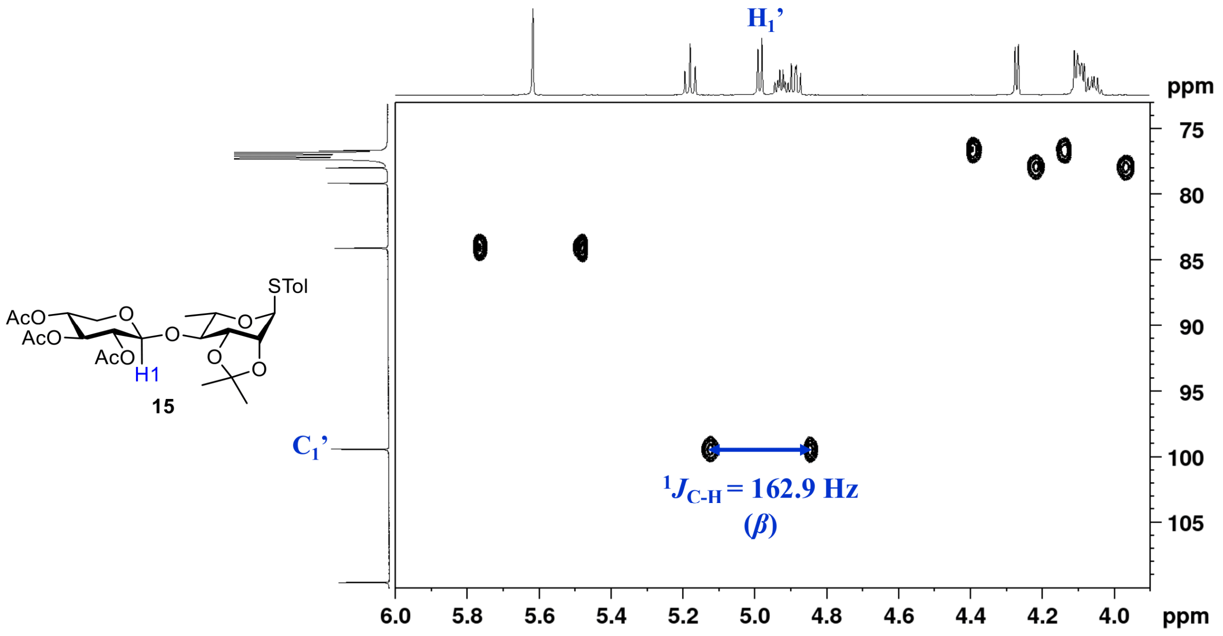

Supplementary Figure S19. 2D-Non-Decoupled HSQC NMR Spectrum of Compound **15**.

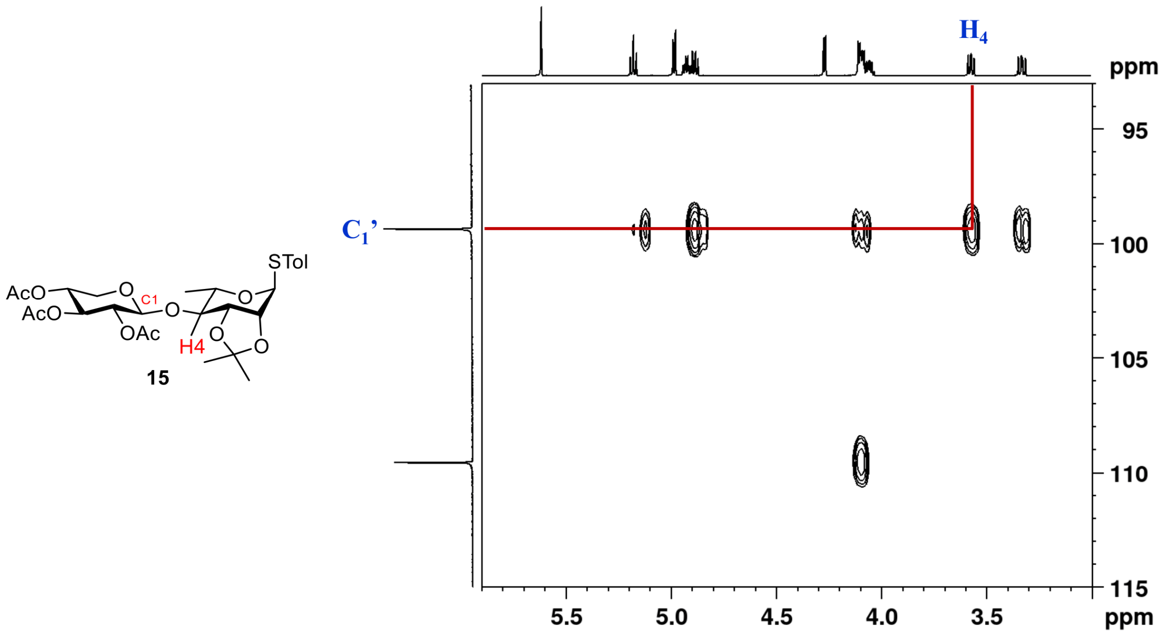

Supplementary Figure S20. 2D-HMBC NMR Spectrum of Compound **15**.

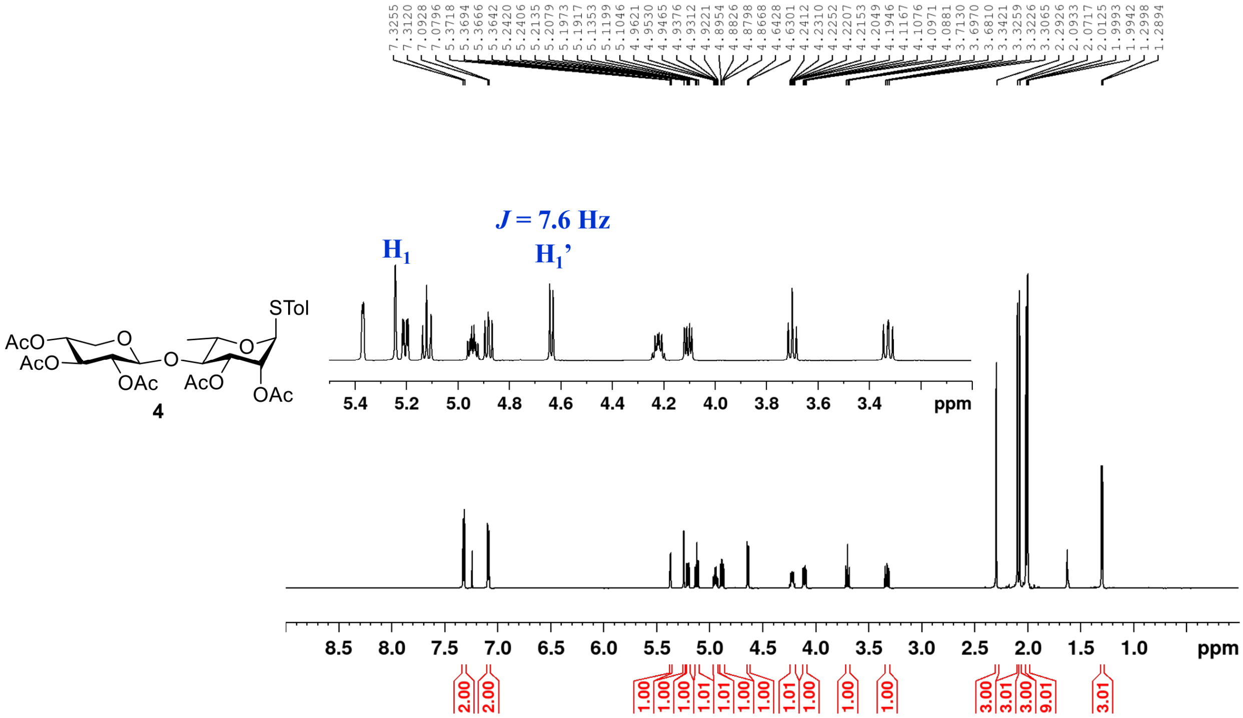

Supplementary Figure S21. ^{1}H NMR Spectrum of Compound **4**.

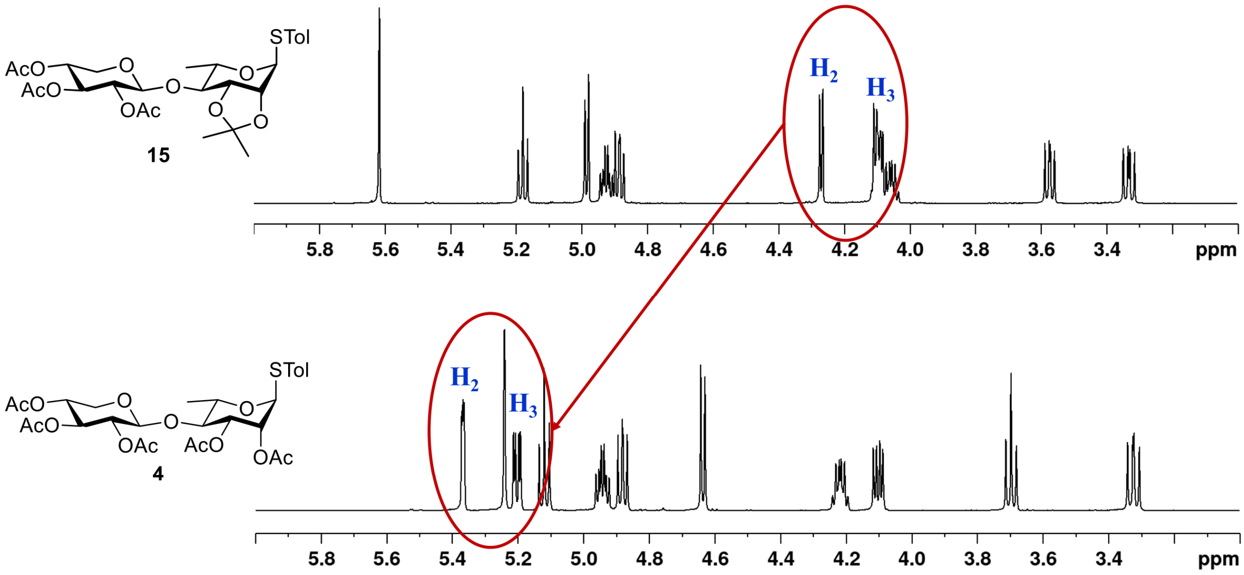

Supplementary Figure S22. Overlay ^{1}H NMR Spectra of **15** (top) and **4** (bottom).

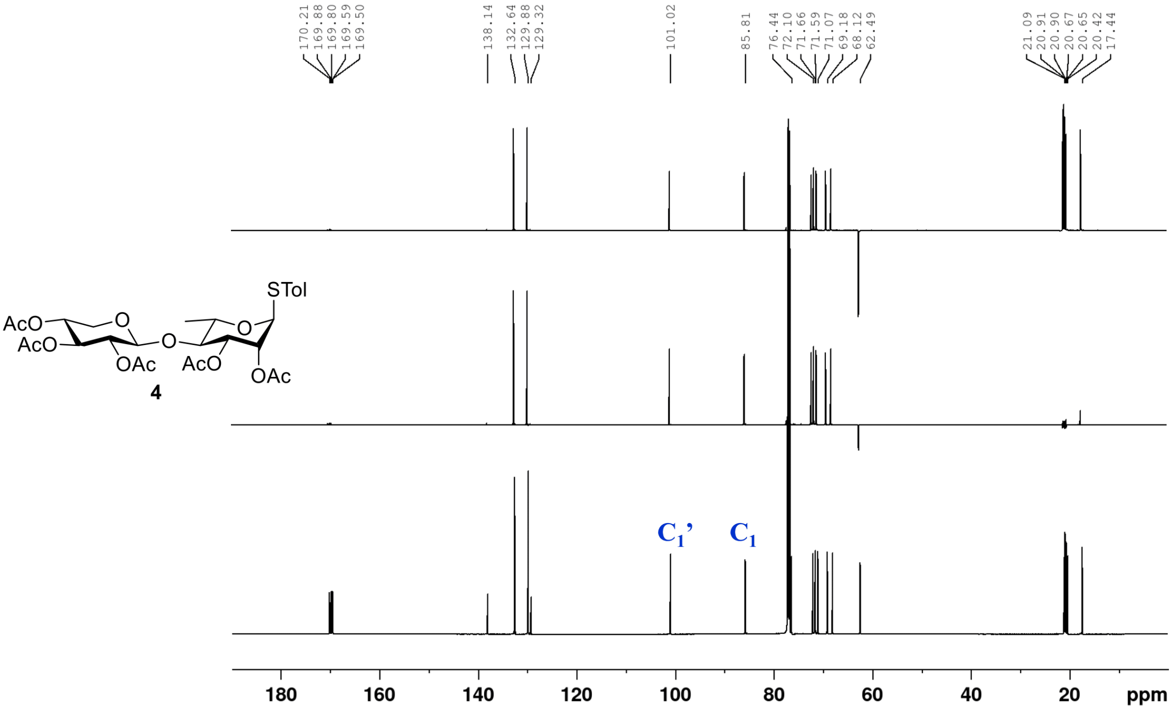

Supplementary Figure S23. ^{13}C NMR Spectrum of Compound **4**.

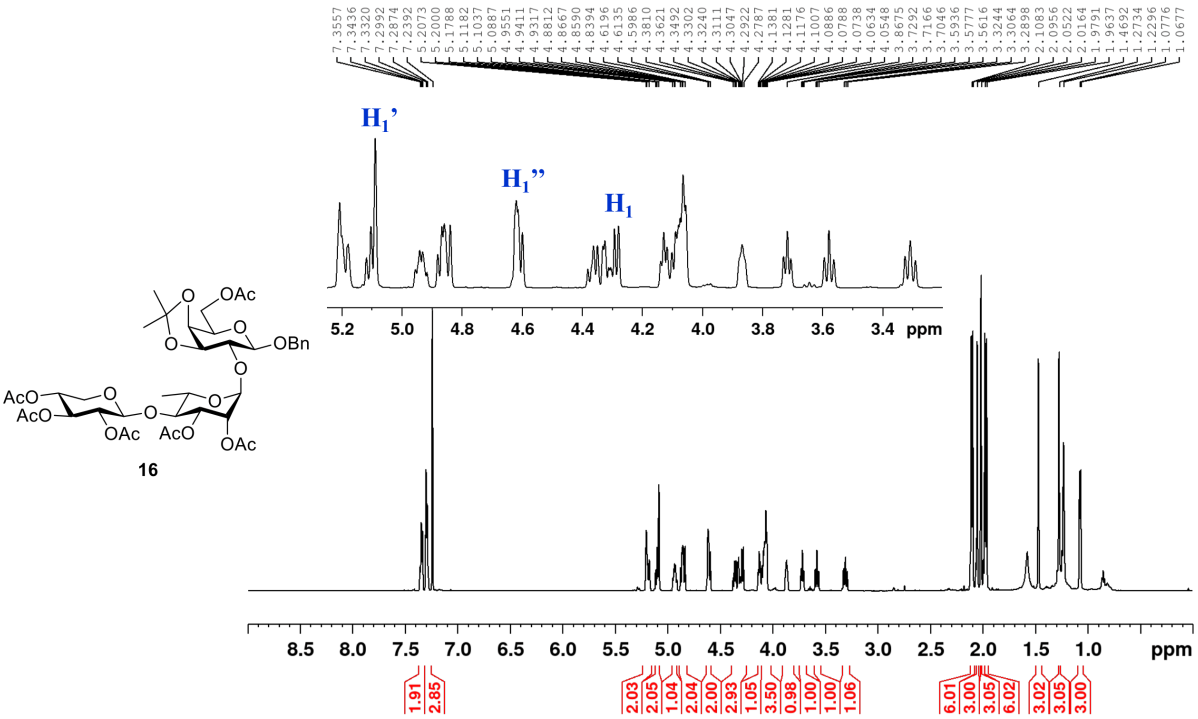

Supplementary Figure S24. ^{1}H NMR Spectrum of Compound **16**.

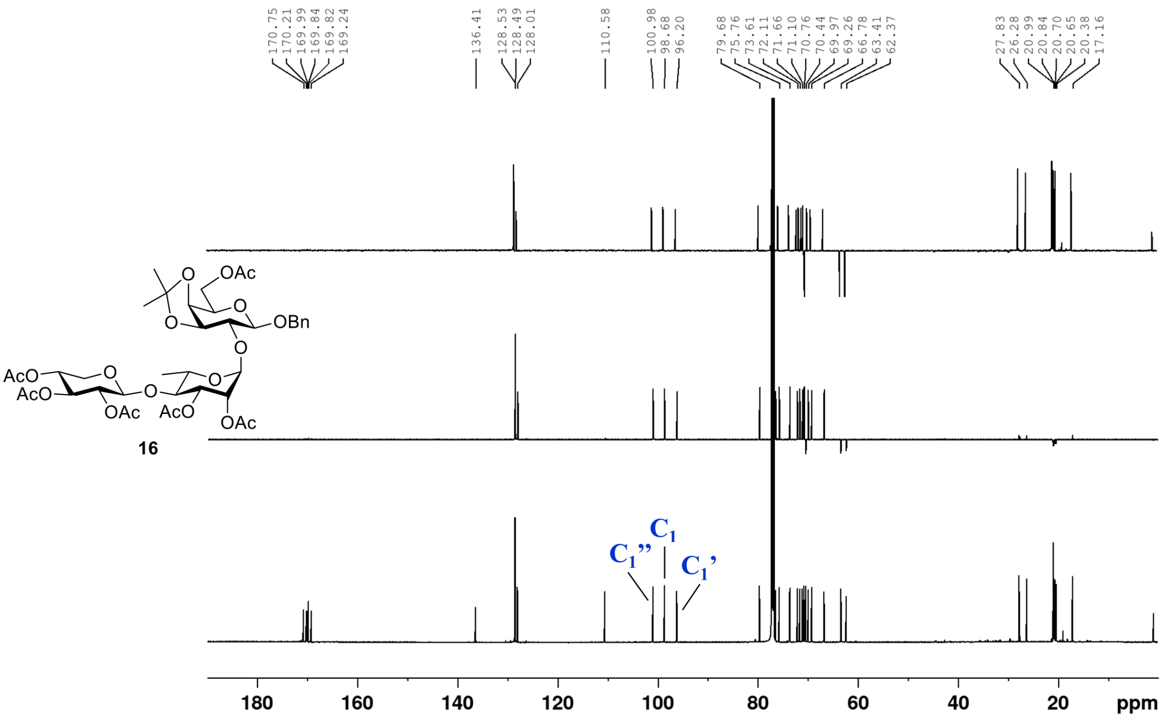

Supplementary Figure S25. ^{13}C NMR Spectrum of Compound **16**.

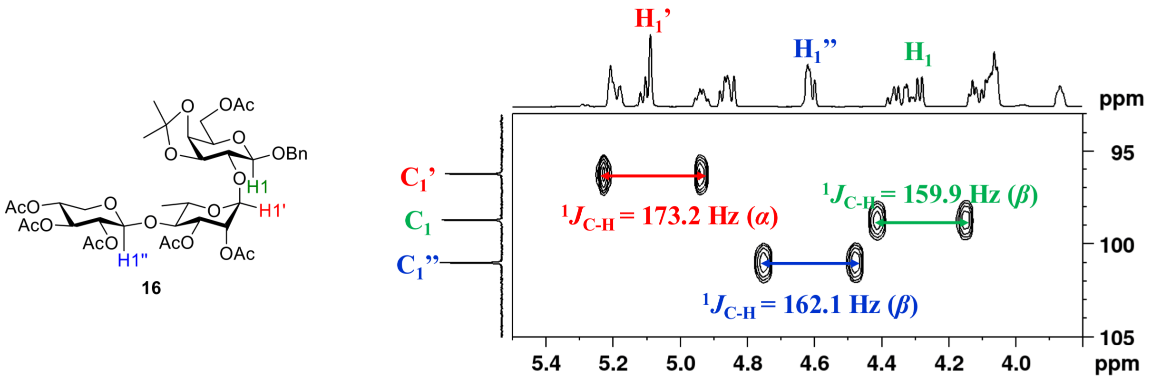

Supplementary Figure S26. 2D-Non-Decoupled HSQC NMR Spectrum of Compound **16**.

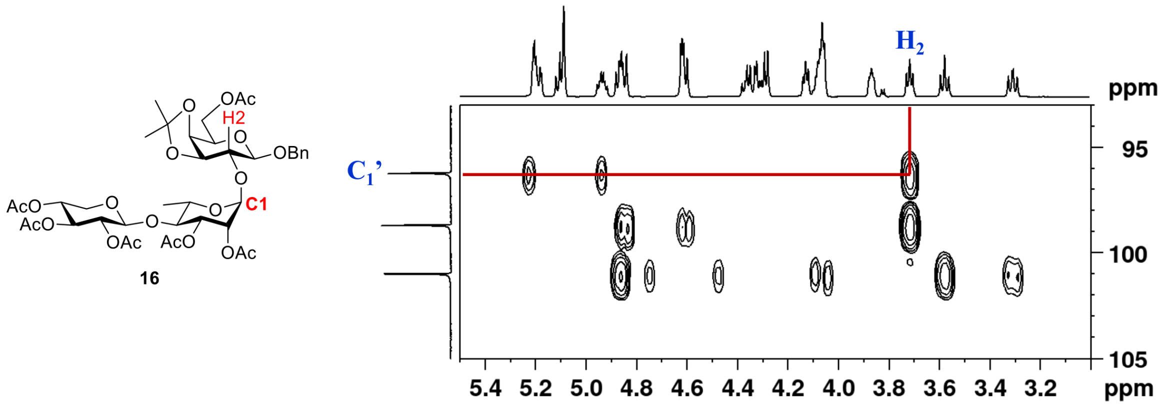

Supplementary Figure S27. 2D-HMBC NMR Spectrum of Compound **16**.

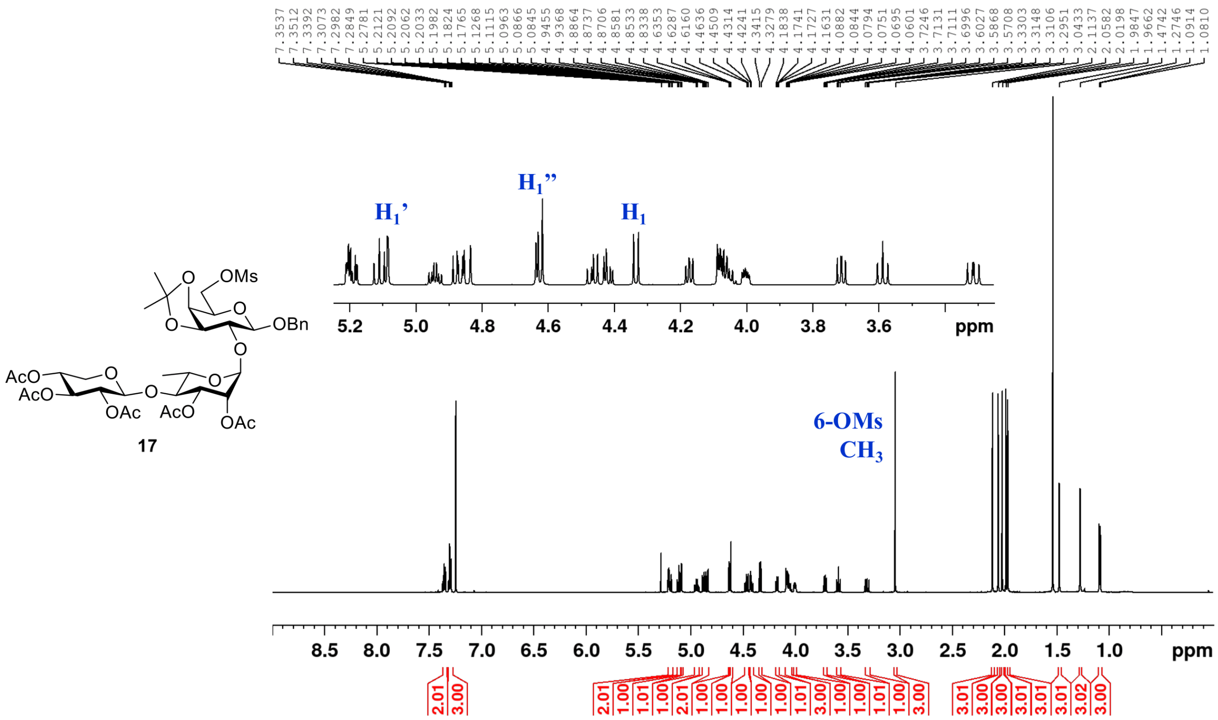

Supplementary Figure S28. ^{1}H NMR Spectrum of Compound **17**.

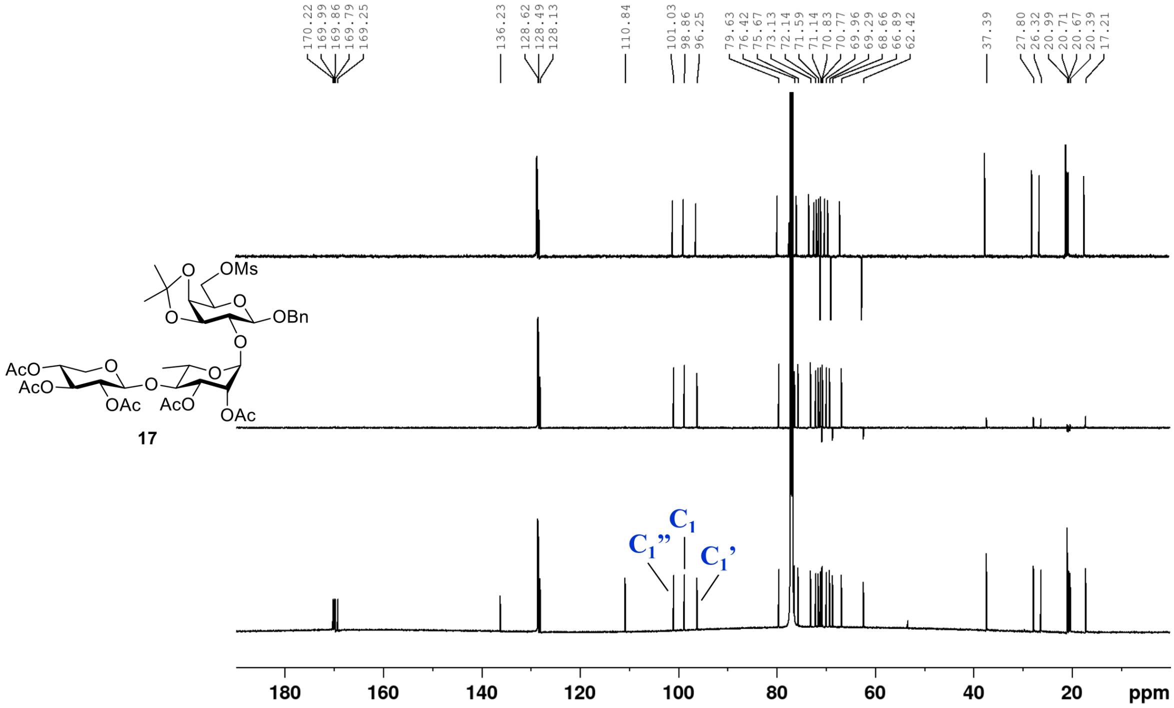

Supplementary Figure S29. ^{13}C NMR Spectrum of Compound **17**.

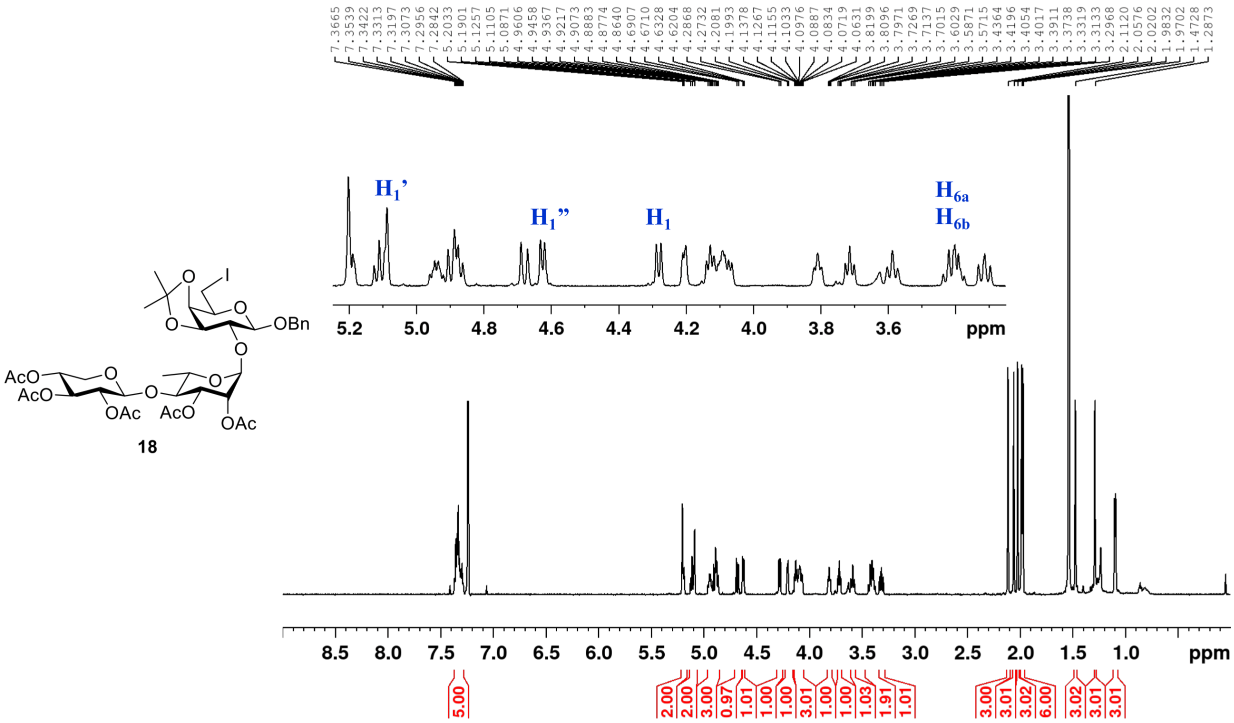

Supplementary Figure S30. ^{1}H NMR Spectrum of Compound **18**.

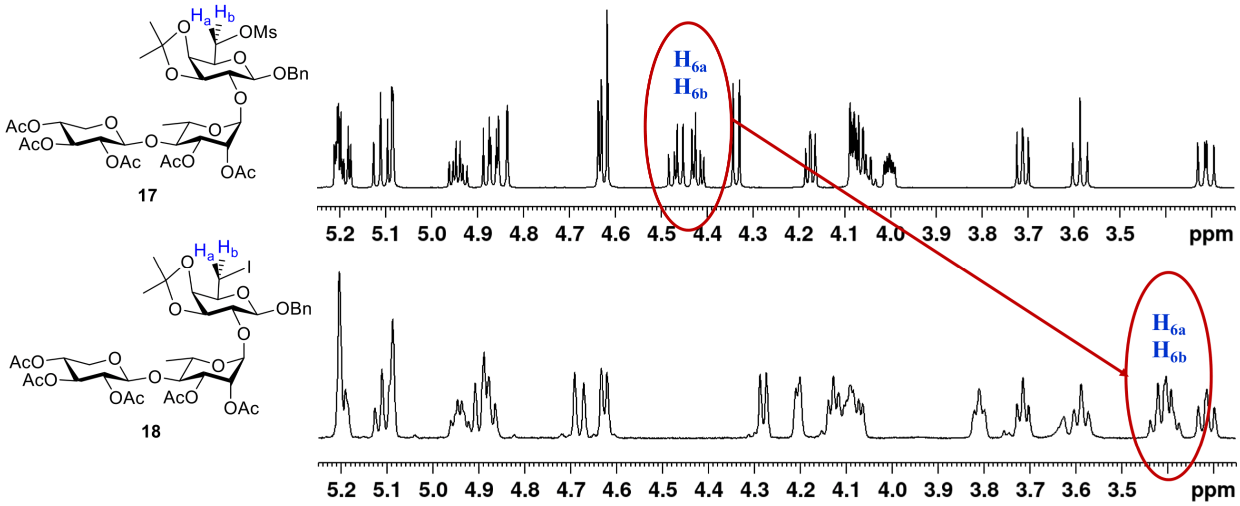

Supplementary Figure S31. Overlay ^{1}H NMR Spectra of **17** (top) and **18** (bottom).

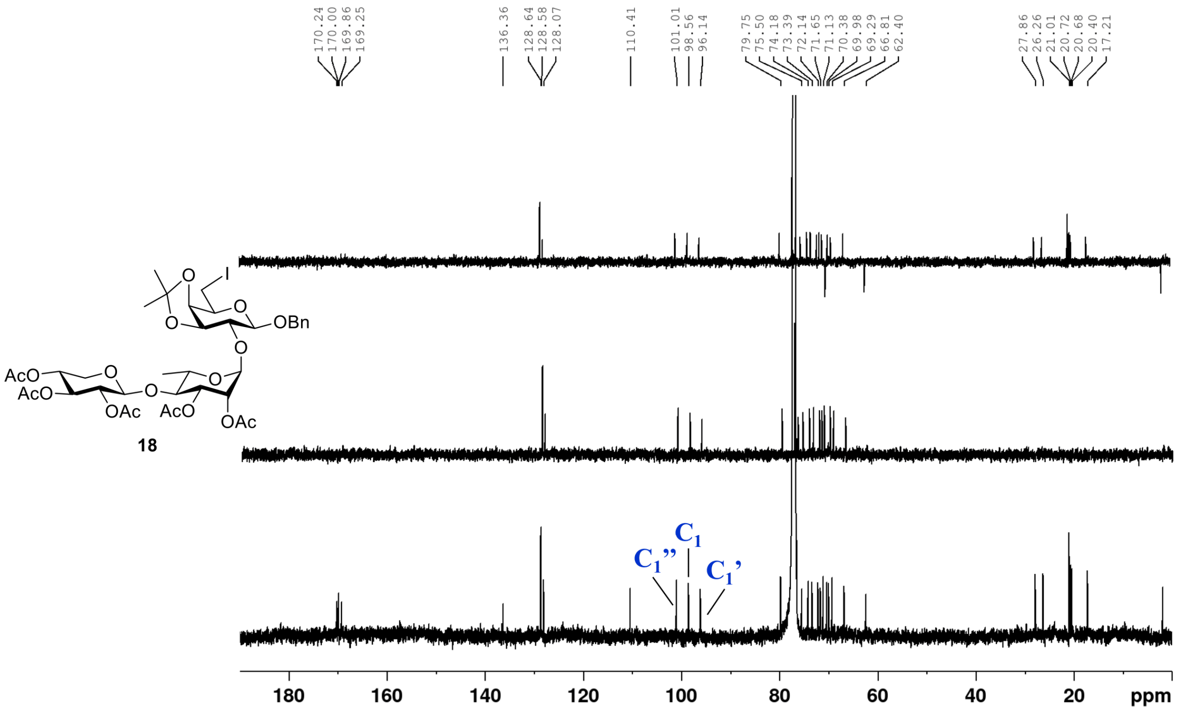

Supplementary Figure S32. ^{13}C NMR Spectrum of Compound **18**.

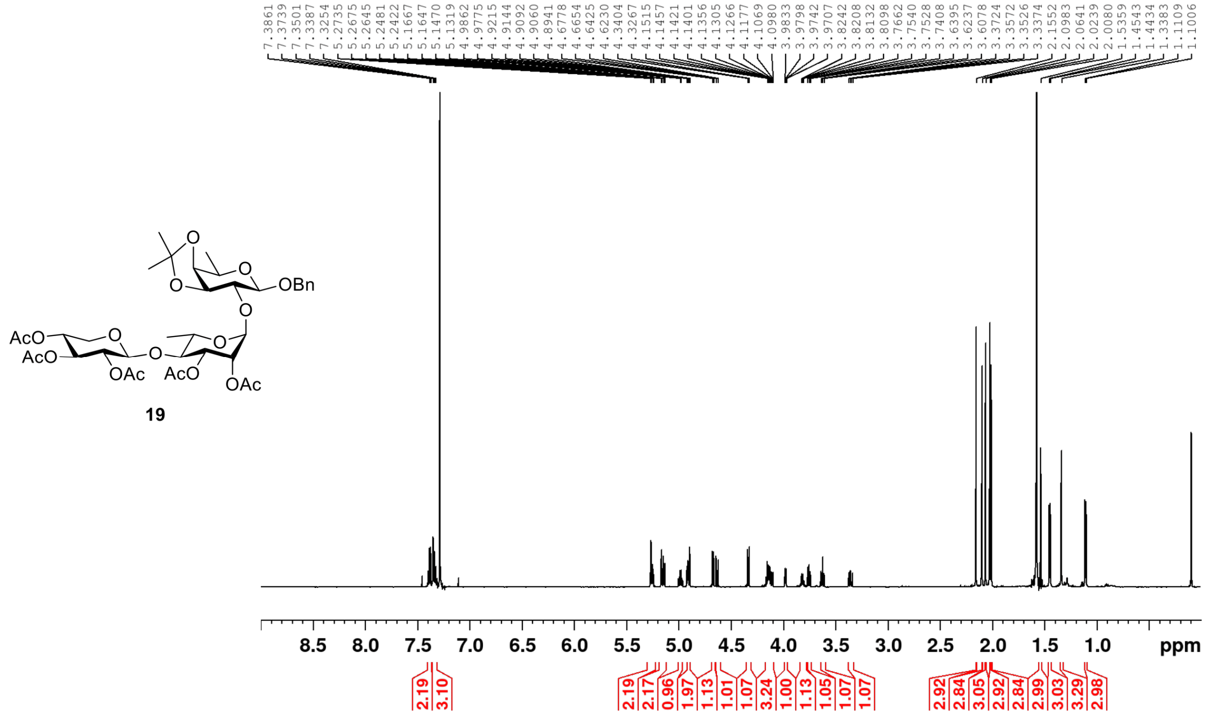

Supplementary Figure S33. ^{1}H NMR Spectrum of Compound **19**.

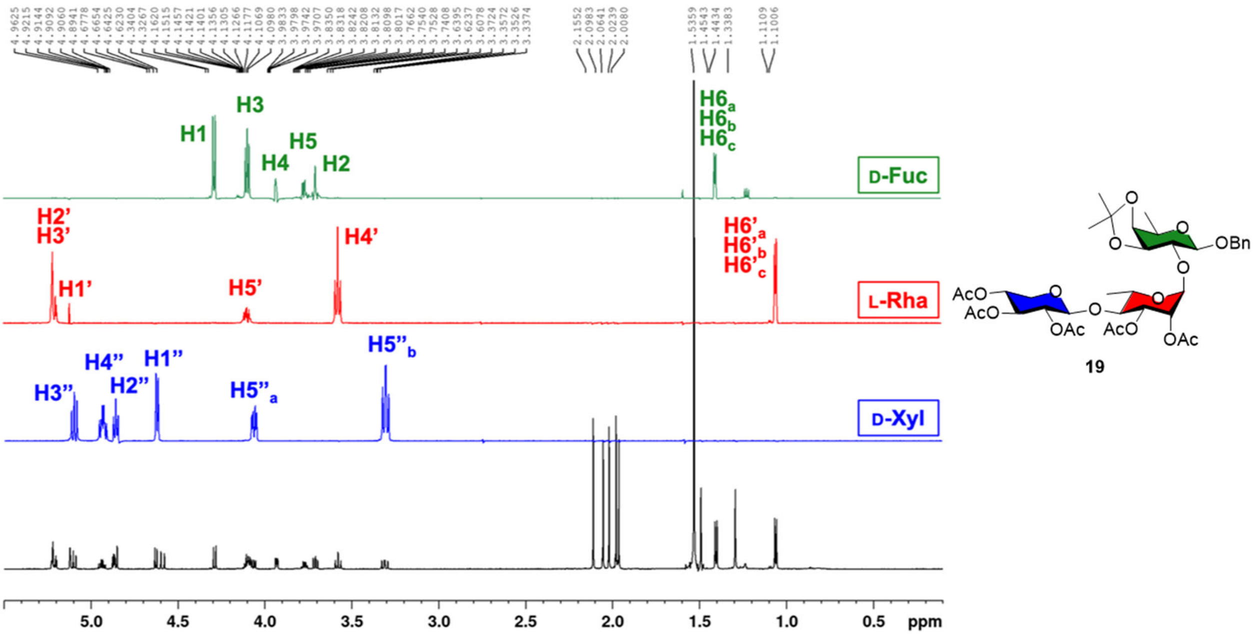

Supplementary Figure S34. Overlay 1D-TOCSY NMR Spectra of Compound **19**.

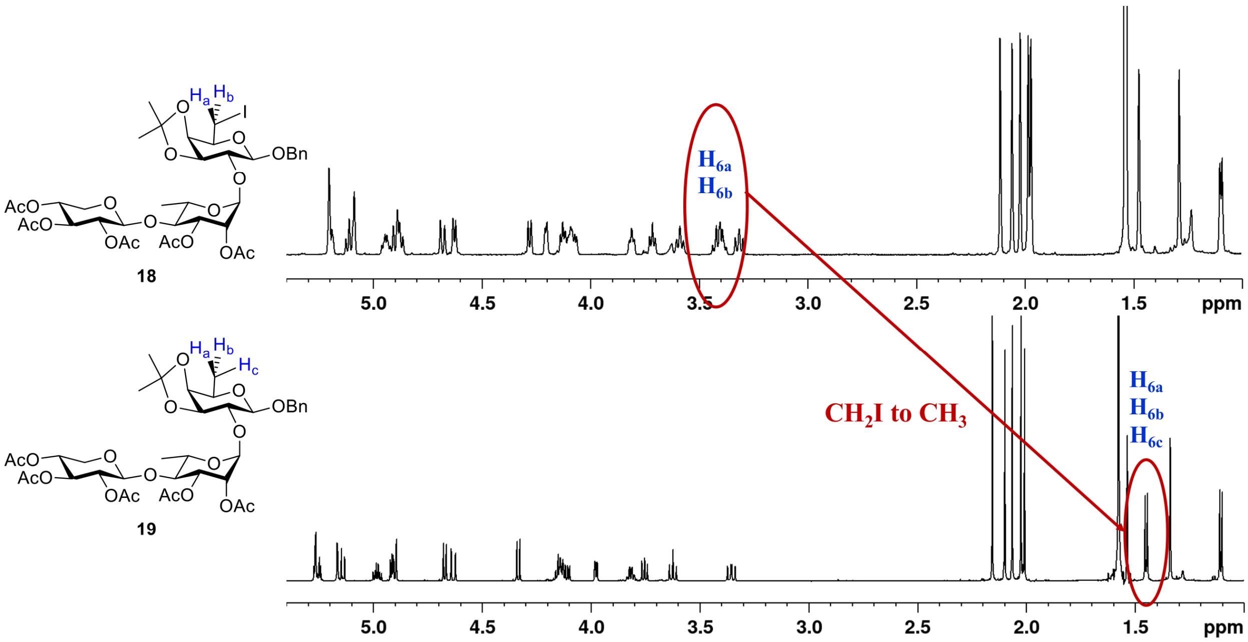

Supplementary Figure S35. Overlay ^{1}H NMR Spectra of **18** (top) and **19** (bottom).

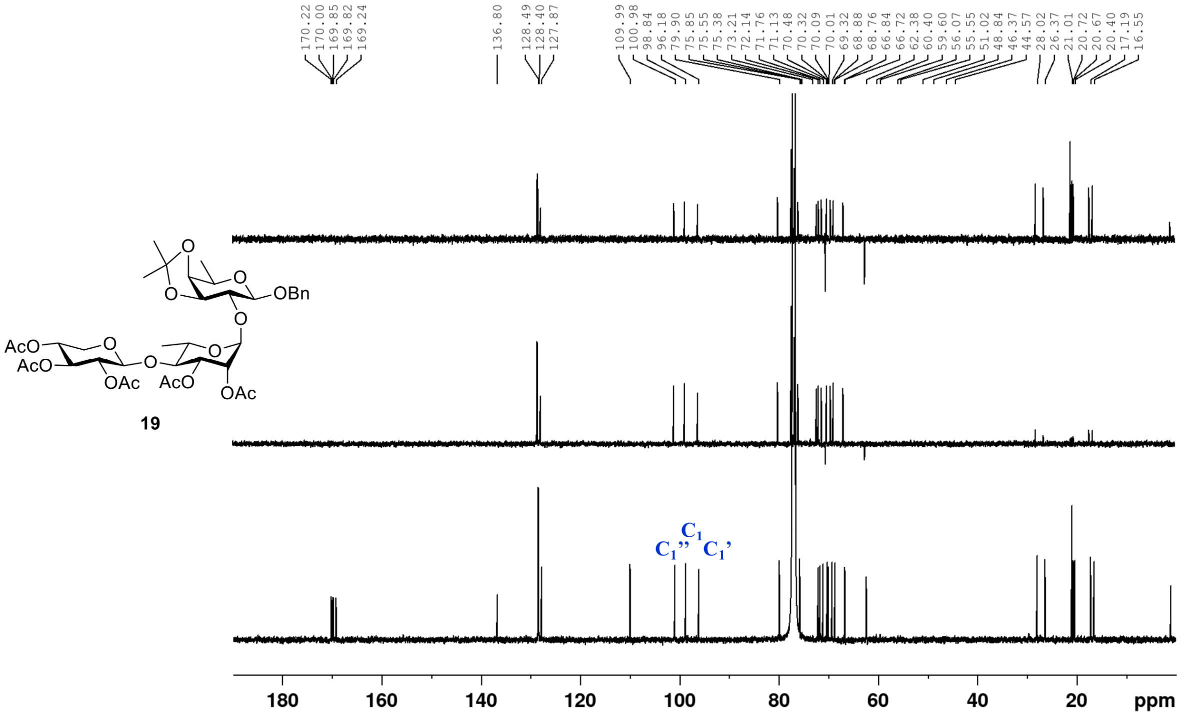

Supplementary Figure S36. ^{13}C NMR Spectrum of Compound **19**.

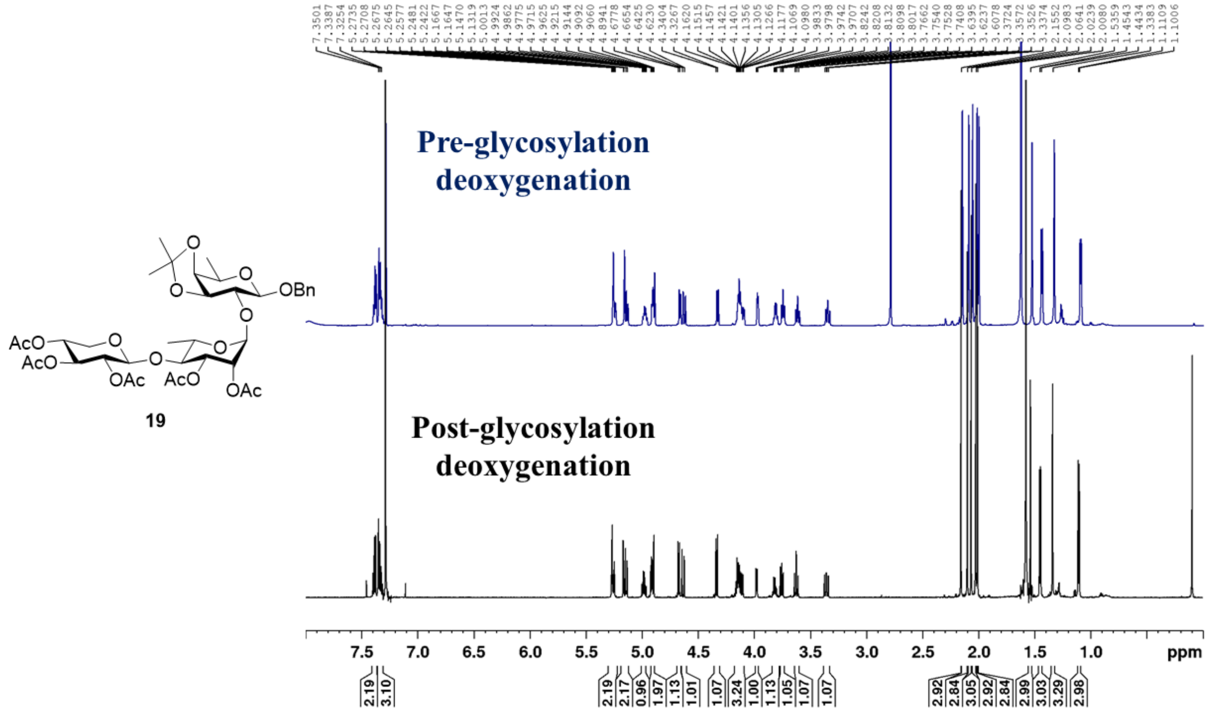

Supplementary Figure S38. Comparison of ^{1}H NMR Spectrum for Compound **19** obtained from pre-glycosylation (top) and post-glycosylation (bottom) strategies.

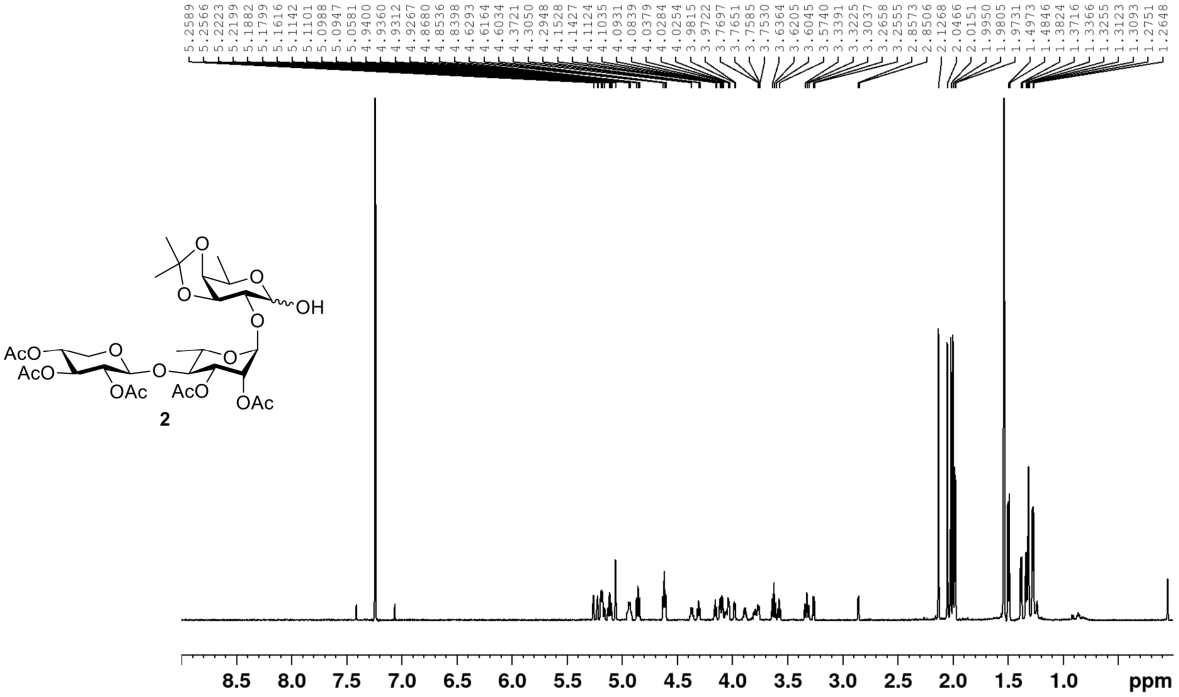

Supplementary Figure S38. ^{1}H NMR Spectrum of Compound **2**.

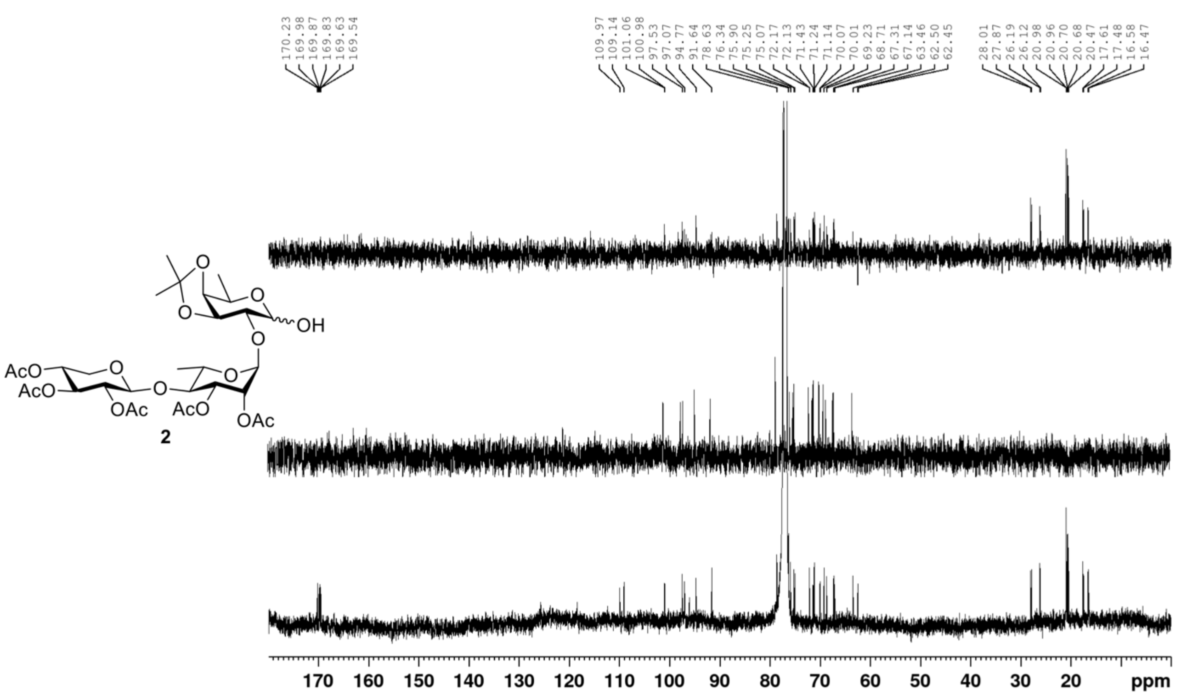

Supplementary Figure S39. ^{13}C NMR Spectrum of Compound **2**.
